# Integrated transcriptomics and molecular docking identify hub genes and statin regulators in *Helicobacter pylori*-associated gastric mucosal pathogenesis

**DOI:** 10.3389/fcimb.2026.1688009

**Published:** 2026-02-13

**Authors:** Minglin Zhang, Xue He, Xuelin Zhao, Ting Cai, Xiaoming Liu, Fen Wang

**Affiliations:** 1 Health Management Medicine Center, The Third Xiangya Hospital, Central South University, Changsha, Hunan, China; 2 Department of Gastroenterology, The Third Xiangya Hospital, Central South University, Changsha, Hunan, China; 3 Nursing Department, The Third Xiangya Hospital, Central South University, Changsha, Hunan, China; 4 Department of Gastroenterology, Hunan Provincial People’s Hospital, The First Affiliated Hospital of Hunan Normal University, Changsha, Hunan, China; 5 Hunan Provincial University Key Laboratory of the Fundamental and Clinical Research on Functional Nucleic Acid, Changsha Medical University, Changsha, Hunan, China

**Keywords:** atorvastatin, *Helicobacter pylori*, hub genes, molecular docking, simvastatin, TPCA-1

## Abstract

**Background:**

*Helicobacter pylori* (*H. pylori*), a spiral Gram-negative bacterium colonizing gastric mucosa, damages epithelial cells, driving chronic gastritis, peptic ulcers, and gastric cancer. Mechanisms of its malignant transformation remain unclear.

**Methods:**

We identified differentially expressed genes (DEGs) and hub genes, pathway enrichment analysis, transcription factors (TFs), small-molecule drug and molecular docking prediction using gastric epithelial cells (GECs) infected with *H. pylori* strain 26695 and clinical sample datasets.

**Results:**

*H. pylori* infection of GECs, multiple signaling pathways were activated, including the p53, MAPK, TNF, IL-17, PI3K-Akt and NF-κB signaling, DNA replication, Apoptosis, Cell cycle, Necroptosis. A total of 107 co-expressed DEGs were identified *in vitro* and *in vivo*. Using Cytoscape, 20 hub genes were prioritized: TNF, CXCL8, NFKBIA, IRF1, ICAM1, TNFAIP3, BIRC3, CXCL1, ITGAM, RELB, CXCL2, CCL20, NFKB2, EGR1, CDKN1A, IRAK2, JAK1, NFKBIE, TRAF1, and JUNB. These hub genes were enriched in NF-κB, TNF and IL-17 signaling, and responses to bacterial pathogens or lipopolysaccharide. Further analysis identified 9 upregulated TFs: VDR, SPI1, ETS2, STAT1, E2F1, PML, ETS1, BRCA1, MYC, and 4 downregulated TFs: ING4, CEBPD, HSF1, JUN. To explore potential therapeutic interventions, we performed small-molecule drug prediction and molecular docking for hub genes revealed: Simvastatin: Linked to CCL20, NFKBIA, and ICAM1. Atorvastatin: Associated with CDKN1A, ICAM1, and TNF. TPCA-1: Targeting JAK1.

**Conclusion:**

These findings provide a theoretical foundation for further investigation into the molecular mechanisms underlying *H. pylori*-related diseases.

## 1 Introduction

*Helicobacter pylori (H. pylori)*, a spiral Gram-negative bacterium that colonizes the gastric mucosa, damages epithelial cells and drives the development of chronic gastritis, peptic ulcers, and gastric cancer (GC) (Arnold et al., 2020; Malfertheiner et al., 2014). It was discovered in 1982 by Barry Marshall and Robin Warren, who later received the Nobel Prize for their groundbreaking work (Pincock, 2005; Warren and Marshall, 1983). This bacterium infects approximately 50% of the global population and is classified as a Group 1 carcinogen by the World Health Organization, making it one of the primary causative agents of GC (Chmiela et al., 2017). Its ability to persist in the harsh acidic environment of the stomach and evade host immune responses underscores its role as a critical driver of gastrointestinal pathology.

During *H. pylori* infection, its virulence factors play critical roles in disease progression. Gastric mucosal biopsies and PCR identified the key *H. pylori* virulence factors:cagA, vacAs2m2, vacAs1m1, vacAs1m2, oipA, babA, dupA, cagE, iceA1 and iceA2 (Barhoine et al., 2025). Additional studies describe other virulence factors such as NapA, GGT (γ-glutamyl transpeptidase), and HtrA (Clyne and Ó Cróinín, 2025). Our prior research revealed distinct pathogenic roles of these factors, *H. pylori* and VacA induce the expression of TRAF1 and TNFAIP3 (Xiao et al., 2025; Zhang et al., 2025c). In gastritis studies: MMP-10 promotes *H. pylori* colonization and inflammation (Lv et al., 2019), MMP-9 is significantly upregulated in *H. pylori*-infected gastric mucosa, exacerbating inflammation (Zhang et al., 2024a), ANGPTL4 (angiopoietin-like 4) and TINAGL1 (tubulointerstitial nephritis antigen-like 1) facilitate *H. pylori* colonization and gastritis progression (Teng et al., 2023; Xie et al., 2024), In *H. pylori*-associated GC research: *H. pylori* induce ONECUT2 expression, enhancing cancer stem cell-like properties (Lin et al., 2024), *H. pylori* promote hnRNPA2B1 and PABPC1 synergy to drive tumor progression (Yu et al., 2024), *H. pylori* upregulate LRP8, triggering β-Catenin nuclear translocation to accelerate carcinogenesis (Liu et al., 2025), *H. pylori* activate the pseudophosphatase STYX, which inhibits FBXO31, further promoting GC (Liu et al., 2022a). Thus, *H. pylori* drive the expression of host genes, activates oncogenic signaling pathways, and fuels the progression from gastritis to GC.

In this study, we established *H. pylori*-infected GECs (HGC27, AGS, MKN45, and GES-1) and performed RNA sequencing (RNA-seq) to identify co-expressed differentially expressed genes (DEGs). By integrating RNA-seq data from clinical *H. pylori*-infected gastric mucosal samples in the Gene Expression Omnibus (GEO) database, we identified *in vitro* and *in vivo* co-expressed DEGs. Subsequent analyses focused on hub genes screening and validation, exploration of transcription factors (TFs), and prediction of small-molecule inhibitors. This systematic approach provides a theoretical foundation for understanding the gene expression patterns and potential regulatory mechanisms underlying *H. pylori*-induced gastric mucosal diseases.

## 2 Materials and methods

### 2.1 Construction of *H. pylori* infected gastric epithelial cell models and RNA-seq

The GC cell lines (HGC27, AGS, and MKN45) and the normal gastric epithelial cell line GES-1 were purchased from the Cell Bank of the Chinese Academy of Sciences (Shanghai). All experiments were performed using low-passage cells (P_3_–P_5_). Cells were cultured in RPMI-1640 medium (BasalMedia, Shanghai) supplemented with 10% fetal bovine serum (Opcel, Nanjing) and maintained at 37 °C in a humidified incubator with 5% CO_2_. This study utilized the standard *H. pylori* strain ATCC 26695 to infect GECs. The wild-type *H. pylori* strain was kindly provided by the laboratories of Dr. Zou and Dr. Lan at the Third Military Medical University. Previous studies have shown that a multiplicity of infection (MOI) of 100 yields optimal infection efficiency in GECs after 24 hours of infection (Xiao et al., 2025; Zhang et al., 2025c). The major virulence factors of *H. pylori* 26695 include CagA, VacA, and UreA (Liu et al., 2024). We established *H. pylori* 26695-infected models using HGC27, AGS, MKN45, and GES-1 cells (n=3, MOI = 100). After 24 hours of infection, cells were harvested and total RNA was extracted. Sequencing libraries were constructed and subjected to transcriptomic profiling on the Illumina NovaSeq 6000 platform (LC-Bio Technologies, China). Genome-wide annotation and differential expression analysis were performed using thresholds of adjusted P-value < 0.05 and absolute |log_2_ fold change| > 0.585 to identify differentially expressed genes (DEGs). The LC-Bio Technology Co., Ltd. platform was used for DEG screening, validation, and visualization. Key analyses included GO (Ashburner et al., 2000) and KEGG (Kanehisa and Goto, 2000) enrichment analyses, along with graphical representations such as bar plots, Venn diagrams, and lollipop plots to illustrate functional pathways and gene overlaps across cell models.

### 2.2 Download of *H. pylori* infected clinical sample transcriptomic data from GEO

Transcriptomic data from *H. pylori*-infected clinical samples were obtained from the GEO database. Matrix files and platform annotation files were downloaded, followed by conversion of raw data into gene expression profiles. Unannotated genes, non-coding RNAs (ncRNAs), miRNAs, and other non-annotated transcripts were filtered out. After rigorous screening and quality control, the GSE233973 dataset (human gastric mucosa, with 13 *H. pylori* infection samples and 9 control samples) was selected as the *in vivo* training set, and GSE27411(human gastric mucosa, with 6 *H. pylori* infection samples and 6 control samples) served as the *in vivo* validation set. Integrated with RNA-seq data from our *H. pylori*-infected gastric epithelial cell models, the virulence factor knockout data from the GSE60661 and GSE243405 datasets were used for *in vitro* validation of hub genes. subsequent bioinformatic analyses and visualization were performed using R software and the LC-Bio online analysis platform. This combined approach ensures robust cross-validation of molecular signatures associated with *H. pylori* infection. The online databases used in this study are detailed in **Supplementary Table S1**.

### 2.3 Screening of programmed cell death-associated DEGs

Upregulated and downregulated DEGs in *H. pylori* infected samples were screened and intersected with autophagy related genes (371 autophagy-related genes were obtained from The Human Autophagy Database), 38 cuproptosis related genes (Xiao et al., 2024), 1607 ferroptosis-related genes were extracted from FerrDb V3 (Zhou et al., 2025), and 120 PANoptosis related genes (Zhang et al., 2025a) to identify DEGs specifically associated with programmed cell death pathways in *H. pylori* infection.

### 2.4 Identification and validation of hub genes *in vivo* and *in vitro*

In *H. pylori*-infected HGC27, AGS, and MKN45 cells, we identified 560 DEGs. Concurrently, 6,061 DEGs were screened in clinical samples from *H. pylori*-infected patients. Comparative analysis revealed 107 co-expressed DEGs shared between *in vitro* (cell models) and *in vivo* (clinical) datasets. A protein-protein interaction (PPI) network was constructed to explore functional relationships among these genes, with disconnected nodes (isolated genes) removed to focus on key interactions. Using Cytoscape software, the co-expressed DEGs were visualized and analyzed. The MCODE plugin was applied to identify densely connected subnetworks, while the CytoHubba plugin with the Degree algorithm prioritized hub genes based on network centrality. Integrating these computational results with our prior research, we identified the Top 20 hub genes, which represent critical regulators of *H. pylori*-induced molecular perturbations in both cellular and clinical contexts. This multi-step approach bridges *in vitro* and *in vivo* findings, ensuring robust identification of hub genes driving *H. pylori*-associated pathogenesis. hub genes were validated *in vivo* using the clinical dataset GSE27411 and *in vitro* in *H. pylori*-infected GES-1 cells.

### 2.5 Functional enrichment analysis

Enrichment analyses GO and KEGG were performed on hub genes using R packages (e.g., clusterProfiler, enrichplot), identifying key signaling pathways potentially regulated by these genes.

### 2.6 Screening of TFs regulating hub genes

The TRRUST database (version 2), a curated resource for transcriptional regulatory interactions, was used to analyze regulatory relationships between hub genes and TFs (Han et al., 2015). hub genes were input into TRRUST to construct a transcriptional regulatory network, and candidate TFs were extracted. A visualization of TF-hub genes regulatory interactions was generated. Next, TFs were intersected with DEGs from the GSE233973 dataset to identify differentially expressed transcription factors (DETFs). Finally, the expression levels of these TFs were validated using the following public datasets: the integrated datasets GSE27411 and GSE233973 (for *H. pylori* infection). We further employed R packages—including limma, corrplot, Hmisc, and reshape2—to calculate Pearson and Spearman correlation coefficients, thereby assessing the correlations between TFs and ETS1 in the *H. pylori* infection datasets (GSE27411 and GSE233973).

### 2.7 Screening and prediction of small-molecule drugs targeting hub genes

The Connectivity Map (CMap), a tool for exploring relationships between genes, small-molecule drugs, and diseases (as previously described in our studies), was utilized to identify potential therapeutic agents targeting hub genes (Lamb et al., 2006). By inputting the hub genes (all upregulated in our analysis) into CMap, we prioritized small-molecule inhibitors with correlation scores < -99, where negative scores indicate antagonistic effects on hub genes expression. Subsequently, the 2D and 3D chemical structures of these candidate drugs were retrieved and visualized using PubChem (Kim et al., 2016), facilitating structural analysis for future drug optimization or repurposing studies.

### 2.8 Molecular docking

Molecular docking studies how small molecule ligands and biological macromolecules recognize and bind (Ferreira et al., 2015). Its core goals are predicting the ligand’s optimal binding site and conformation on the target protein, and estimating binding affinity. Ligand structures are typically sourced from PubChem, protein structures from the RCSB PDB (Burley et al., 2021). Docking is performed using the CB-Dock platform, which employs a deep learning-based scoring function (Liu et al., 2022b). A lower (more negative) Vina score indicates stronger binding; ≤ -5.0 kcal/mol suggests potential activity. Results include 3D binding diagrams for interaction analysis.

### 2.9 RNA extraction, RT-qPCR and Western blot

The protocols for RNA extraction, RT-qPCR and WB have been described in detail in our previous studies (Zhang et al., 2024b, 2025, 2025). The primers used in this study are listed in **Supplementary Table S2**. The primary and secondary antibodies employed are specified in **Supplementary Table S3**.

### 2.10 Clinical samples and immunohistochemistry

Human gastric mucosal tissue samples were collected from the Department of Pathology, Third Xiangya Hospital of Central South University, during two periods: from January 2019 to March 2020. *H. pylori* infection was diagnosed using the C13/C14 urea breath test or histopathological examination. A positive result was confirmed when at least two of the aforementioned tests yielded consistent findings. Both *H. pylori*-negative (n=22) and *H. pylori*-positive gastric mucosal tissues (n=41) were collected, with pathological diagnoses including chronic gastritis and atrophic gastritis.

This study was conducted in accordance with the principles of the Declaration of Helsinki and was approved by the Human Experimentation Committee (Ethics Committee) of Third Xiangya Hospital, Central South University (ethical approval number: Fast|21170). Written informed consent was obtained from all participating patients.

Immunohistochemical images were acquired using a microscope (Nikon, Japan). Staining intensity was scored as follows: 0 (no staining), 1 (weak staining), 2 (moderate staining), and 3 (strong staining). The proportion of stained cells was also scored: 0% = 0, 1–24% = 1, 25–49% = 2, 50–74% = 3, and 75–100% = 4. The final immunohistochemistry score was calculated by multiplying the intensity score by the proportion score, resulting in a range from 0 (lowest) to 12 (highest). The immunohistochemical analysis, data analysis, and certain experimental procedures performed in this study were conducted with reference to previously established methods (Zhang et al., 2024b, 2025, 2025).

### 2.11 Statistical analysis

Data analysis was conducted in R software (v4.5.1). Data analysis and visualization were performed using GraphPad Prism 9.0 software, a two-tailed Student’s t test or one-way ANOVA. Statistical significance computed by the Wilcoxon test was annotated by the number of stars. The results were expressed as the means ± standard deviations (SDs), and all analyses were performed at least in triplicate. Significance was set at *p < 0.05, **p < 0.01, ***p < 0.001, or ****p < 0.0001; ns, not significantly different.

## 3 Results

### 3.1 Identification of DEGs in *H. pylori* infected

**Figure 1:**
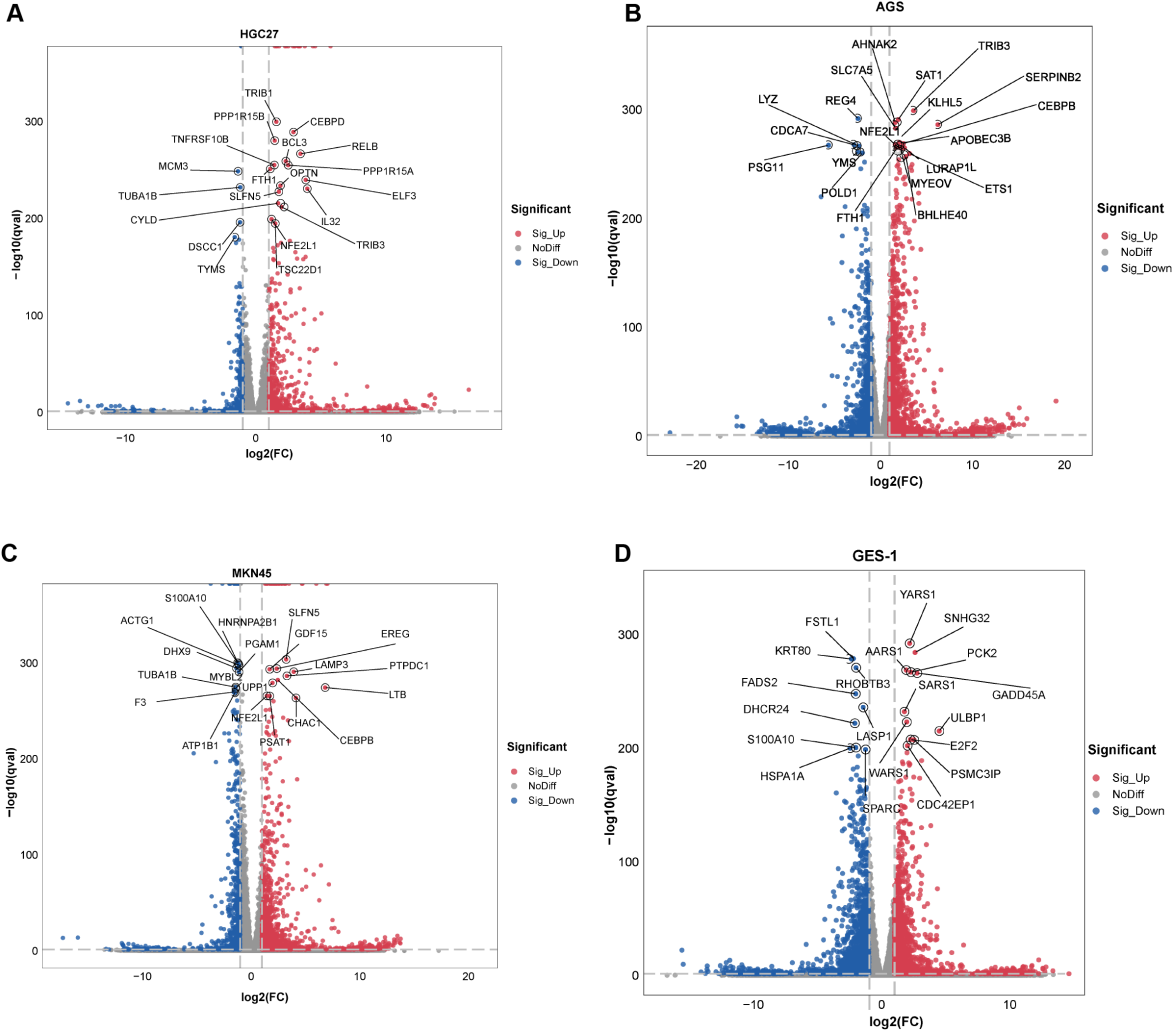
Volcano plots of DEGs. **(A–D)** RNA-seq analysis of DEGs in HGC27, AGS, MKN45, and GES-1 cells infected with *H. pylori* (n = 3, MOI = 100, 24 h). Volcano plots display the top 20 DEGs, with the x-axis representing log_2_ (fold change, FC) and the y-axis representing −log_10_ (adjusted *P*-value, qval). Thresholds: |log_2_FC| ≥ 0.585 and adjusted *P*-value < 0.05. Red: significantly upregulated genes; blue: significantly downregulated genes; gray: non-significant genes.

To investigate *H. pylori*-infected gastric mucosal epithelial cell lines (AGS, HGC27, MKN45 and GES-1), RNA-seq was performed to analyze DEGs using the thresholds |log_2_FC| ≥ 0.585 and P-value < 0.05. Volcano plots were generated to display the top 20 DEGs. In HGC27, the top three DEGs were TRIB1, CEBPD and PPP1R15B (**Figure 1A**; **Supplementary Figure S1A**). In AGS, the top three DEGs were TRIB3, REG4 and SAT1 (**Figure 1B**; **Supplementary Figure S1B**). In MKN45, the top three DEGs were SLFN5, S100A10 and HNRNPA2B1 (**Figure 1C**; **Supplementary Figure S1C**). In GES-1, the top three DEGs were YARS1, SNHG32 and FSTL1 (**Figure 1D**; **Supplementary Figure S1D**).

To further identify highly significant DEGs, stricter thresholds were applied (|log_2_FC| ≥ 4 and P-value ≈ 0). In AGS, the significant DEGs included CXCL8, HKDC1, UPP1, GDF15, MMP1, PTPRH, ATF3, IL32 and DMBT1 (**Supplementary Figure S2A**). In MKN45, the significant DEGs were INHBE, TNFRSF9, IL20RB, TRIB3 and IL32 (**Supplementary Figure S2B**). In HGC27, the significant DEGs were CCL2, TNFAIP3 and CFB (**Supplementary Figure S2C**). In GES-1, ASNS was the sole significant DEG (**Supplementary Figure S2D**). A Venn diagram analysis revealed three overlapping DEGs (WARS1, VEGFA and SDC4) shared among AGS, HGC27 and MKN45 (**Supplementary Figure S2E**). These results demonstrate that the DEGs identified in *H. pylori*-infected GECs vary across cell lines, likely due to cellular heterogeneity. Nonetheless, this study provides a foundation for screening and validating *H. pylori* infection-related DEGs in diverse gastric epithelial models.

### 3.2 Functional enrichment analysis of DEGs

**Figure 2:**
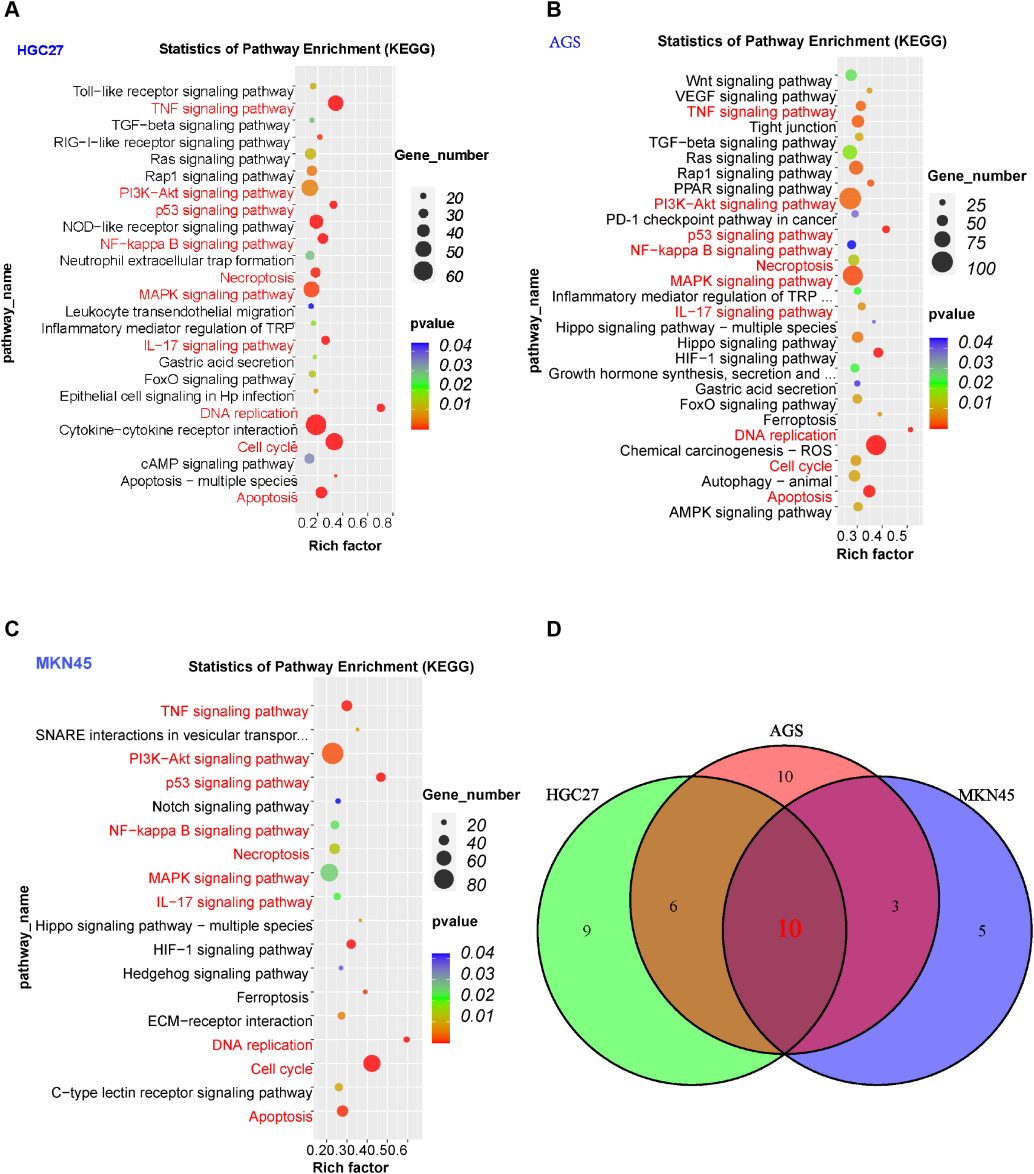
Pathway enrichment analysis of DEGs. **(A–C)** KEGG pathway enrichment analysis of *H. pylori*-infected gastric epithelial cells (HGC27, AGS, MKN45). Pathways marked in red are shared across the three cell lines. Circle size represents the number of genes enriched in each pathway, and color intensity (closer to red) indicates lower *P*-values (greater statistical significance). **(D)** Venn diagram showing 10 overlapping pathways enriched in all three gastric epithelial cell models.

*H. pylori* infection activates multiple signaling pathways, inducing inflammatory responses in GECs and promoting the progression of gastritis to cancer. To explore this, we performed enrichment analysis on the DEGs identified in RNA-seq. KEGG pathway analysis revealed significant enrichment of the p53 signaling pathway, DNA replication, Apoptosis, MAPK signaling pathway, TNF signaling pathway, PI3K-Akt signaling pathway, IL-17 signaling pathway, Cell cycle, Necroptosis, and NF-kappa B signaling pathway in *H. pylori*-infected GECs (HGC27, AGS, and MKN45) (**Figures 2A–C**). Venn diagram analysis identified 10 shared pathways across these cell lines (**Figure 2D**). Further inclusion of *H. pylori*-infected normal gastric mucosal epithelial cells (GES-1) revealed 6 common pathways: p53 signaling pathway, DNA replication, Apoptosis, MAPK signaling pathway, PI3K-Akt signaling pathway, and Cell cycle (**Supplementary Figures S3A, B**).

The DEGs identified in this study include several genes with direct or indirect roles in metabolic regulation. Their heterogeneity across cell lines aligns with the diverse metabolic demands and stress responses of different gastric epithelial models. Focusing on these metabolic hubs could uncover novel mechanisms by which *H. pylori* manipulate host metabolism to establish infection or promote disease progression. To investigate this, we performed metabolic pathway enrichment analysis on *H. pylori*-infected GECs. Venn diagram analysis of metabolic pathways in HGC27, AGS, and MKN45 identified 3 shared metabolic pathways: Glycine, serine and threonine metabolism, Alanine, aspartate and glutamate metabolism, and Terpenoid backbone biosynthesis (**Supplementary Figures S4A, C, D**). Upon further inclusion of *H. pylori*-infected normal gastric mucosal epithelial cells (GES-1), only the alanine, aspartate and glutamate metabolism remained enriched (**Supplementary Figures S5A, B**). These findings highlight both shared and cell-specific metabolic and signaling adaptations in *H. pylori*-infected GECs, providing critical insights into the interplay between *H. pylori*-driven metabolic reprogramming, inflammation, and carcinogenesis.

### 3.3 DEGs associated with programmed cell death in *H. pylori* infection

**Figure 3:**
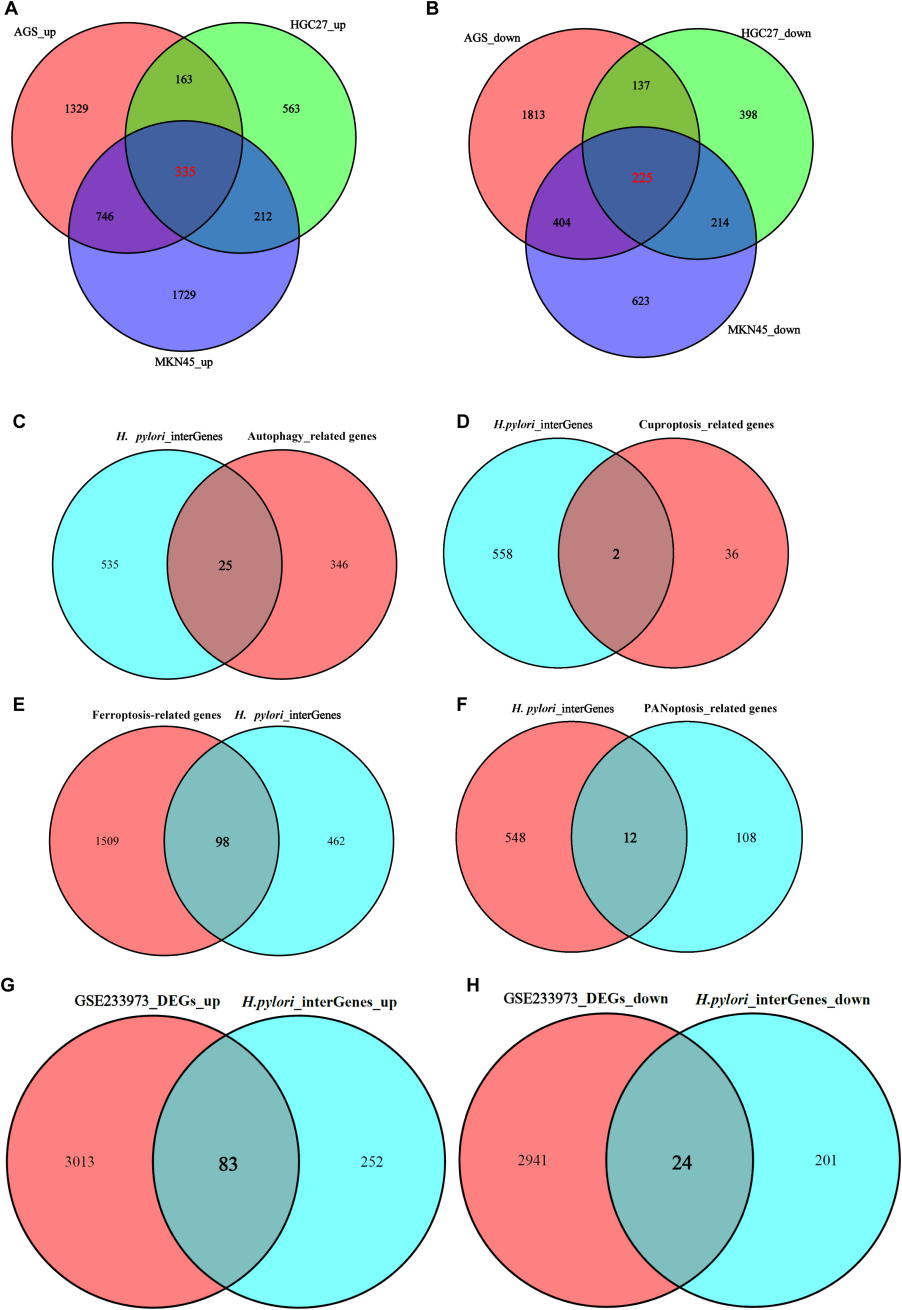
Screening of DEGs and expression of PCD-related genes. **(A)** 335 co-upregulated DEGs shared among *H. pylori*-infected HGC27, AGS, and MKN45 cells. **(B)** 225 co-downregulated DEGs shared among *H. pylori*-infected HGC27, AGS, and MKN45 cells. **(C)** Overlapping genes between co-expressed DEGs and autophagy-related genes in *H. pylori* infection. **(D)** Overlapping genes between co-expressed DEGs and cuproptosis-related genes in *H. pylori* infection. **(E)** Overlapping genes between co-expressed DEGs and ferroptosis-related genes in *H. pylori* infection. **(F)** Overlapping genes between co-expressed DEGs and PANoptosis-related genes in *H. pylori* infection. **(G)** Overlapping genes between co-upregulated DEGs in *H. pylori* infection and upregulated DEGs in the GSE233973 dataset. **(H)** Overlapping genes between co-downregulated DEGs in *H. pylori* infection and downregulated DEGs in the GSE233973 dataset.

Our previous work described 21 cell death modes. This study focuses on exploring the relationship between DEGs and genes linked to autophagy, cuproptosis, ferroptosis, and PANoptosis. First, we screened DEGs in *H. pylori*-infected GECs. HGC27: 1,273 upregulated DEGs; AGS: 2,573 upregulated DEGs; MKN45: 3,022 upregulated DEGs, 335 shared upregulated DEGs were identified via Venn diagram analysis (**Figure 3A**). HGC27: 974 downregulated DEGs; AGS: 2,579 downregulated DEGs; MKN45: 1,466 downregulated DEGs, 225 shared downregulated DEGs were identified via Venn diagram analysis (**Figure 3B**). Thus, a total of 560 DEGs were identified across *H. pylori*-infected HGC27, AGS and MKN45 cells. Next, we analyzed overlaps between these DEGs and genes associated with specific cell death pathways, autophagy-related genes: 25 overlapping genes (**Figure 3C**); cuproptosis-related genes: 2 overlapping genes (**Figure 3D**); ferroptosis-related genes: 98 overlapping genes (**Figure 3E**); PANoptosis-related genes: 12 overlapping genes (**Figure 3F**). These results highlight the heterogeneity of gene expression across *H. pylori*-infected gastric epithelial cell models. To further investigate *H. pylori*’s pathogenic mechanisms, we analyzed clinical gastric mucosal samples from *H. pylori*-infected patients in the GEO database (dataset GSE233973), identifying: 3,096 upregulated DEGs and 2,965 downregulated DEGs. Overlap analysis with DEGs from *H. pylori*-infected GECs revealed 83 shared upregulated DEGs and 24 shared downregulated DEGs (**Figures 3G, H**). This integrative approach underscores both cell line-specific and clinically relevant molecular signatures of *H. pylori* infection, providing critical insights into its role in programmed cell death and disease progression.

### 3.4 Identification and validation of hub genes in *H. pylori*-infected gastric mucosa *in vivo* and *in vitro*

**Figure 4:**
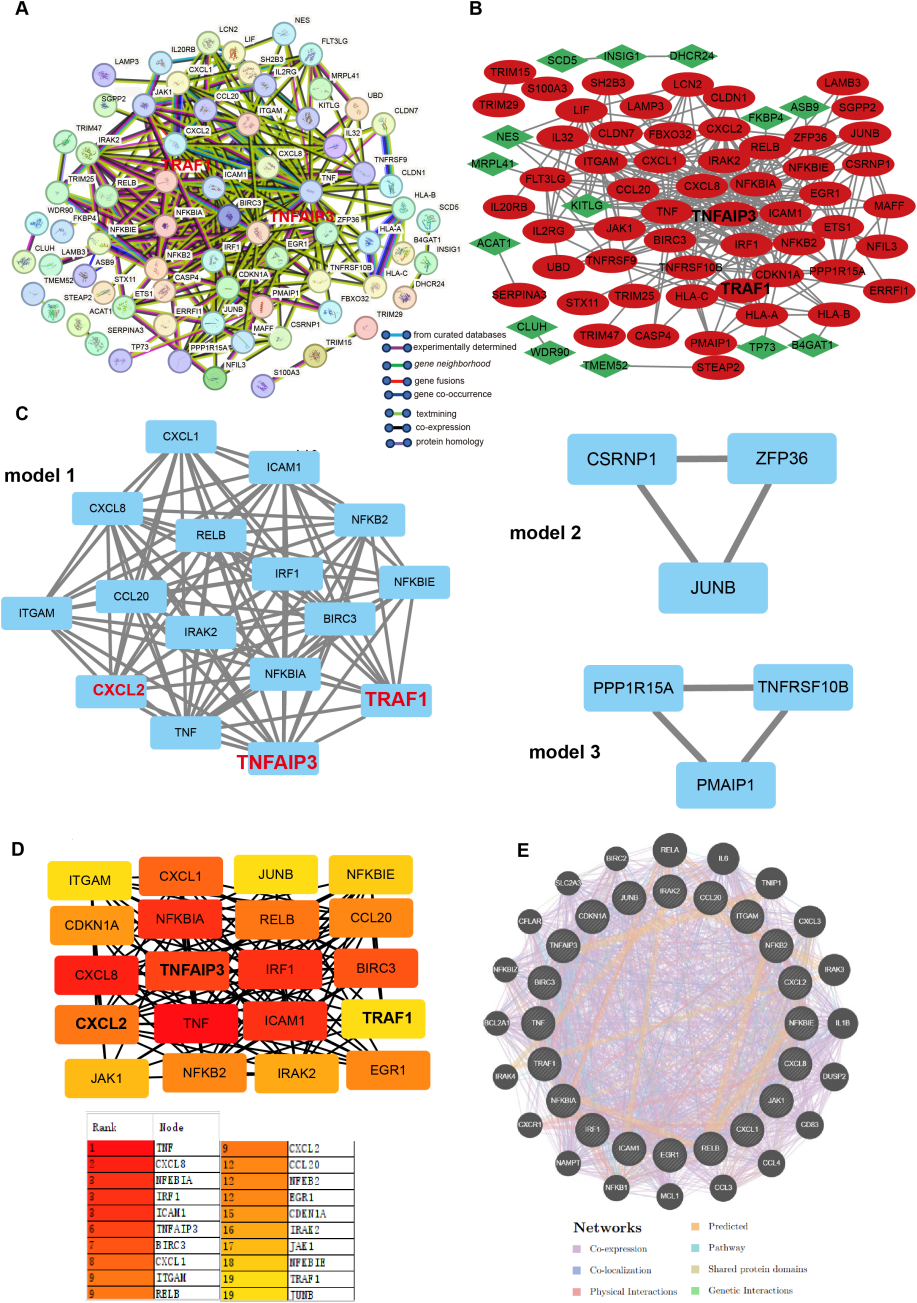
Identification of hub genes. **(A)** PPI network of 107 DEGs derived from the STRING database. Nodes represent distinct genes, and colored lines indicate interaction types between gene pairs. **(B)** Visualization of DEGs in Cytoscape. Red nodes denote upregulated genes, green nodes denote downregulated genes, and connecting lines represent gene-gene interactions. **(C)** Subcluster analysis of DEGs using the MCODE plugin in Cytoscape, revealing three highly interconnected modules (Model 1, 2, 3). Genes highlighted in red (TRAF1, TNFAIP3, CXCL2) were previously identified in our prior studies. **(D)** hub genes identification via the cytoHubba plugin in Cytoscape. Top 20 genes were selected based on the Degree algorithm (degree score ≥ 11). The intensity of red coloration corresponds to higher degree scores, indicating stronger hub characteristics. Genes labeled in bold black (TRAF1, TNFAIP3, CXCL2) are key candidates from our earlier research. **(E)** Functional interaction network of hub genes generated by the GeneMANIA database. Line colors represent specific interaction types (e.g., co-expression, physical interactions).

To further screen hub genes co-expressed in *H. pylori*-infected clinical samples and GECs, we input 107 DEGs into the STRING database, removed disconnected nodes, and constructed a PPI network, medium confidence > 0.4 (**Figure 4A**). The DEGs were visualized using Cytoscape, with upregulated genes depicted in red and downregulated genes in green (**Figure 4B**). Subnetworks of DEGs were generated using the MCODE plugin in Cytoscape, revealing highly interconnected and functionally clustered genes. Notably, the subnetworks highlighted strong associations among TRAF1, TNFAIP3, and CCL2, consistent with our previous findings (**Figure 4C**). hub genes were prioritized using the CytoHubba plugin with the Degree algorithm, identifying 20 hub genes (TNF, CXCL8, NFKBIA, IRF1, ICAM1, TNFAIP3, BIRC3, CXCL1, ITGAM, RELB, CXCL2, CCL20, NFKB2, EGR1, CDKN1A, IRAK2, JAK1, NFKBIE, TRAF1, JUNB) with a degree score ≥ 11 (**Figure 4D**). Finally, the GeneMANIA online tool was employed to construct an interaction network for these hub genes, exploring potential functional relationships such as co-localization, co-expression, shared pathways, and physical interactions (**Figure 4E**). This integrative approach bridges computational predictions with experimental relevance, identifying key molecular players in *H. pylori*-induced gastric pathogenesis.

**Figure 5:**
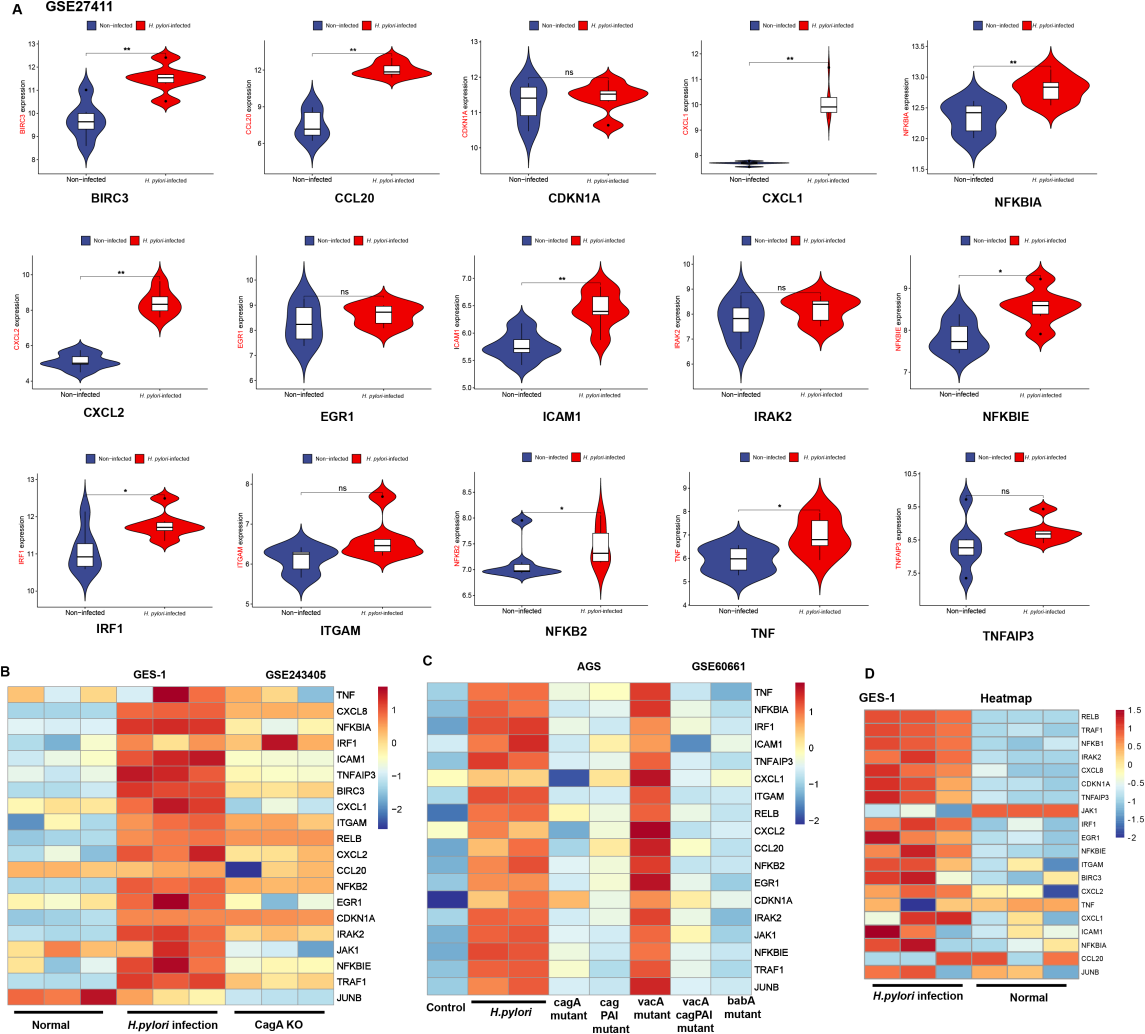
Validation of hub genes. **(A)** Validation of hub genes in the GSE27411 dataset: *H. pylori*-infected *vs*. non-infected groups (*p < 0.05; **p < 0.01; ns: no statistical significance). **(B, C)** Expression heatmaps of hub genes, GES-1 cells: Normal (uninfected) *vs H. pylori*-infected *vs* cagA knockout (KO); AGS cells: Control (uninfected) *vs H. pylori* TN2gf6 strain *vs* cagA mutant *vs* cag PAI mutant *vs* vacA mutant *vs* vacA-cag PAI double mutant *vs* babA mutant. **(D)** Validation of hub genes in *H. pylori*-infected GES-1 cells: *H. pylori*-infection *vs*. Normal.

To further validate the expression of hub genes in *H. pylori*-infected gastric mucosa and GECs, we analyzed clinical samples and cell models. In dataset GSE27411, the expression of BIRC3, CCL20, CXCL1, NFKBIA, CXCL2, ICAM1, NFKBIE, IRF1, NFKB2 and TNF was significantly upregulated in *H. pylori*-infected gastric mucosa (**Figure 5A**). *H. pylori* virulence factors exert distinct regulatory effects on gene expression. By analyzing RNA-seq data from *H. pylori* strains in the GEO database, including those with cagA knockout, cagA mutation, cagPAI mutation, vacA mutation, vacA-cagPAI double mutation, and babA mutation, we found that hub genes were upregulated following *H. pylori* infection, while their expression was downregulated after cagA knockout (**Figure 5B**). Furthermore, hub genes expression decreased following cagA knockout, cagPAI mutation, vacA-cagPAI double mutation, and babA mutation, but increased in the vacA mutant strain (**Figure 5C**). In our established *H. pylori*-infected GES-1 cell model, RNA-seq analysis revealed that most hub genes were upregulated after infection, with the exception of JAK1, which showed downregulated expression (**Figure 5D**). Heatmap visualization of hub genes expression in *H. pylori*-infected GECs (AGS, HGC27, and MKN45) confirmed their upregulation (**Figures S6A–C**). Notably, inconsistencies in hub genes expression across different samples and cell models were observed, likely due to cellular heterogeneity or context-specific regulatory mechanisms. To address this, future studies should incorporate proteomic analysis and multi-omics approaches (e.g., transcriptomics, proteomics, and metabolomics) to reduce model-specific biases and comprehensively unravel the molecular mechanisms driving *H. pylori*-induced pathogenesis. These efforts will provide a robust theoretical foundation for understanding *H. pylori* infection-related disease progression.

### 3.5 Enrichment analysis of hub genes

**Figure 6:**
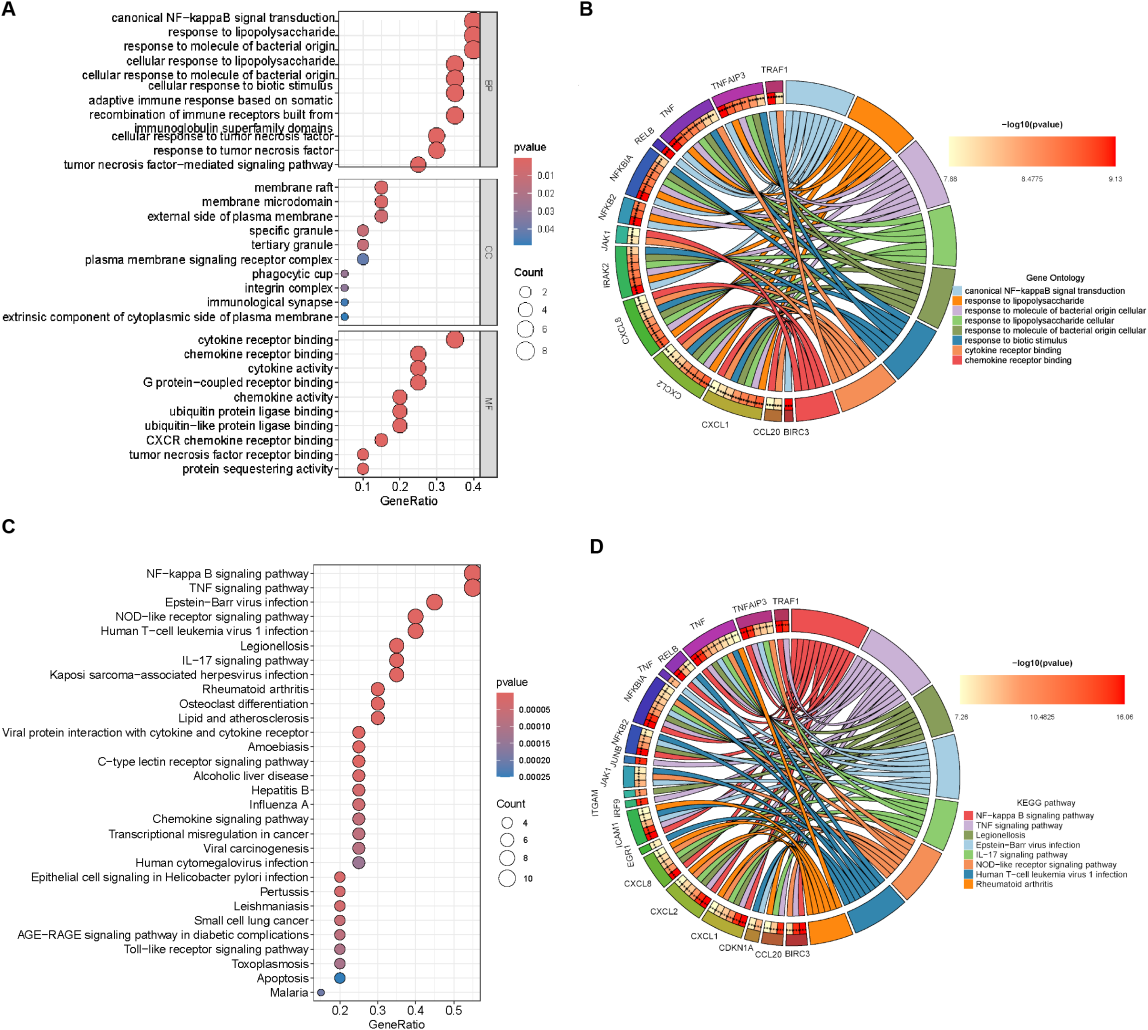
Enrichment analysis of hub genes. **(A)** GO enrichment analysis of DEGs. BP, biological process; CC, cellular component; MF, molecular function. **(B)** Circular visualization of GO enrichment results. The left semicircle represents hub genes (labeled by gene names), while the right semicircle displays enriched GO terms. Colored lines connect genes to their corresponding GO terms, with distinct colors indicating different functional categories. **(C)** KEGG pathway enrichment analysis of DEGs. **(D)** Circular visualization of KEGG enrichment results. The left semicircle represents hub genes (labeled by gene names), while the right semicircle displays enriched KEGG pathways. Colored lines connect genes to their associated pathways, with distinct colors indicating specific pathway categories.

To explore the signaling pathways potentially regulated by the hub genes, we performed GO (Gene Ontology) and KEGG (Kyoto Encyclopedia of Genes and Genomes) enrichment analyses. GO enrichment analysis revealed that the hub genes are primarily involved in the following biological processes and molecular functions: Canonical NF-κB signal transduction; Response to lipopolysaccharide (LPS); Response to molecule of bacterial origin; Cellular response to lipopolysaccharide; Cellular response to molecule of bacterial origin; Cellular response to biotic stimulus; Cytokine receptor binding; Chemokine receptor binding (**Figures 6A, B**). KEGG pathway enrichment analysis identified the following key pathways associated with the hub genes: NF-κB signaling pathway; TNF signaling pathway; Legionellosis; Epstein-Barr virus infection; IL-17 signaling pathway; NOD-like receptor signaling pathway; Human T-cell leukemia virus 1 infection; Rheumatoid arthritis (**Figures 6C, D**). These findings suggest that the hub genes play pivotal roles in innate immunity, inflammatory responses, and host-pathogen interactions, providing mechanistic insights into *H. pylori*-driven gastric pathogenesis.

### 3.6 Prediction and validation of transcription factors regulating hub genes

**Figure 7:**
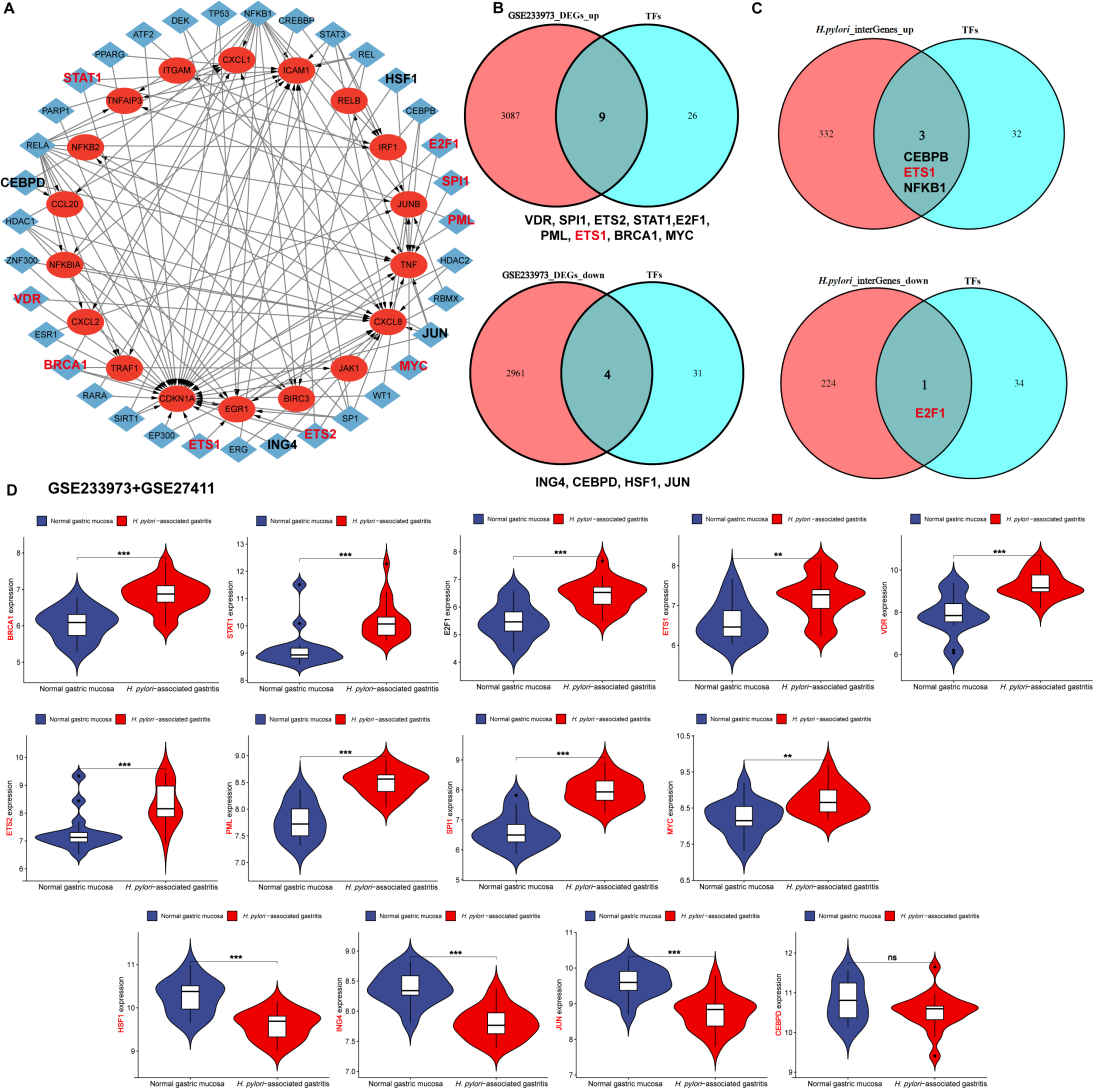
Screening and validation of transcriptional regulators of hub genes. **(A)** Regulatory network visualization of hub genes and TFs. Red circles represent Hub genes, blue rectangles denote TFs. Upregulated TFs are highlighted with thick red borders, downregulated TFs with thick black borders. Black arrows indicate regulatory relationships (TFs → hub genes). **(B)** Overlapping DEGs between TFs and the GSE233973 dataset, identifying 9 upregulated and 4 downregulated DEGs. **(C)** Overlapping genes between DEGs in *H. pylori*-infected gastric epithelial cells (HGC27, AGS, MKN45) and TFs, revealing 3 upregulated and 1 downregulated gene. **(D)** Visual validation of the 13 candidate TFs in the GSE27411 dataset. **p < 0.01; ***p < 0.001. ns, not significantly different.

Using the TRRUST database (version 2), we analyzed TFs potentially regulating the hub genes and identified 35 candidate TFs (**Figure 7A**). To pinpoint TFs specifically altered by *H. pylori* infection, we integrated analysis of DEGs from *H. pylori*-infected gastric mucosa and identified: 9 upregulated TFs: VDR, SPI1, ETS2, STAT1, E2F1, PML, ETS1, BRCA1, MYC. 4 downregulated TFs: ING4, CEBPD, HSF1, JUN (**Figure 7B**). In *H. pylori*-infected GECs, we observed: 3 upregulated TFs: CEBPB, ETS1 and NFKB1; 1 downregulated TF: E2F1 (**Figure 7C**). Integrated analysis revealed that ETS1 was the only TF significantly upregulated in both *H. pylori*-infected gastric mucosa and epithelial cells (**Figures 7A–C**). To validate these findings, we performed an integrated analysis of the clinical datasets GSE233973 and GSE27411 and examined the expression of TFs. The upregulated TFs included VDR, SPI1, ETS2, E2F1, PML, ETS1, RCA1, STAT1, and MYC, while the downregulated TFs were ING4, CEBPD, HSF1, and JUN (**Figure 7D**). These results highlight ETS1 as a key transcription factor that is consistently activated across *H. pylori* infection models, suggesting its central role in regulating hub gene networks during infection. Therefore, in the merged dataset of GSE233973 and GSE27411, further analysis revealed a positive correlation between the expression of ETS1 and that of the hub genes (**Supplementary Figure S7**).

### 3.7 Prediction of small-molecule drugs targeting hub genes

**Figure 8:**
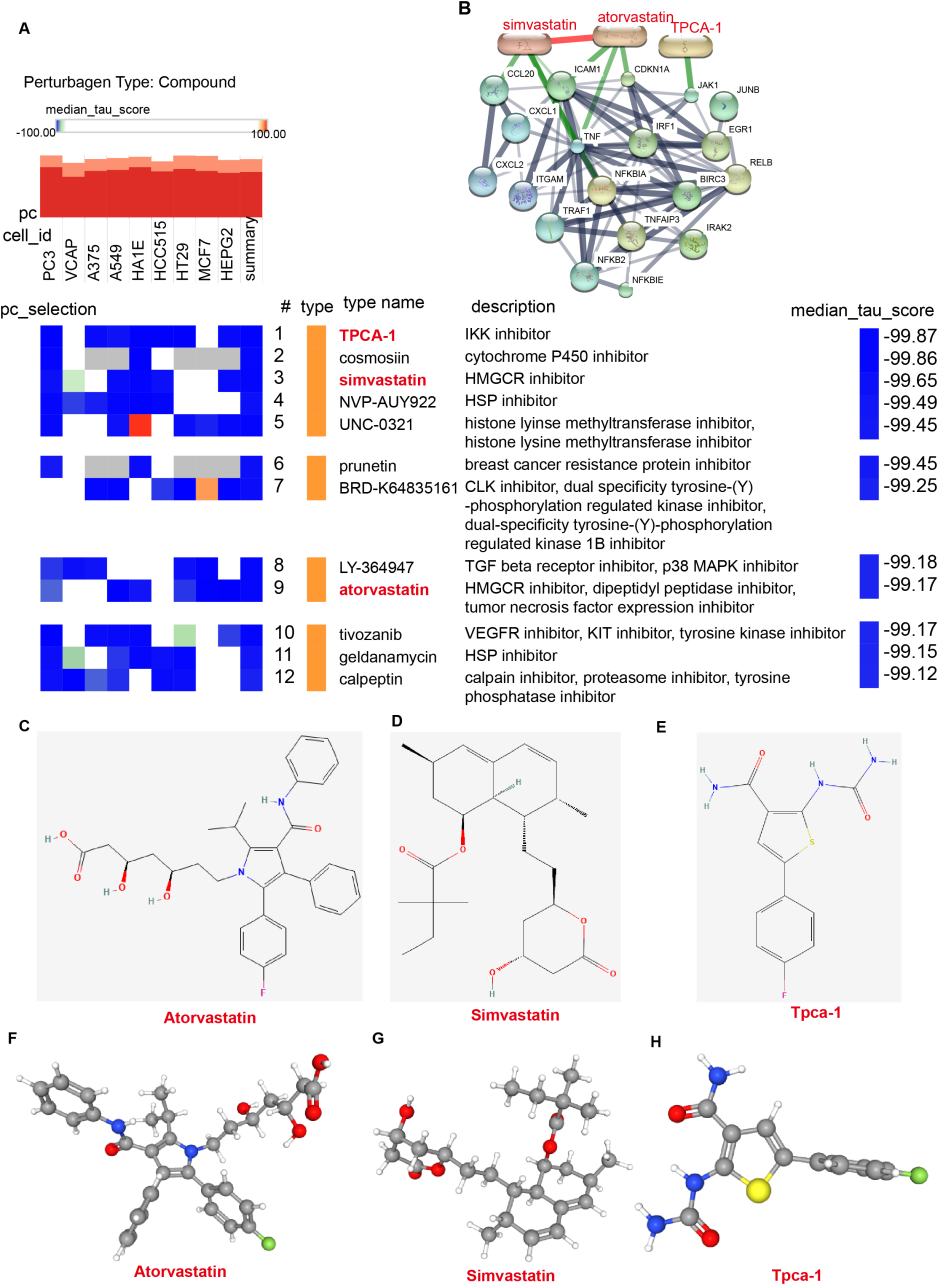
Prediction of small-molecule drugs targeting hub genes. **(A)** Heatmap displaying 12 small-molecule drugs identified via Connectivity Map (CMap) analysis (score < −99) across 10 cell lines. Perturbagen class: compound. The heatmap includes drug class and description. **(B)** PPI network diagram of the 12 compounds and hub genes. Simvastatin, Atorvastatin, and TPCA-1 exhibit strong associations with hub genes. **(C–E)** Chemical structures of the three candidate compounds: Simvastatin, Atorvastatin, and TPCA-1. **(F–H)** Three-dimensional (3D) molecular structures of Simvastatin, Atorvastatin, and TPCA-1.

**Figure 9:**
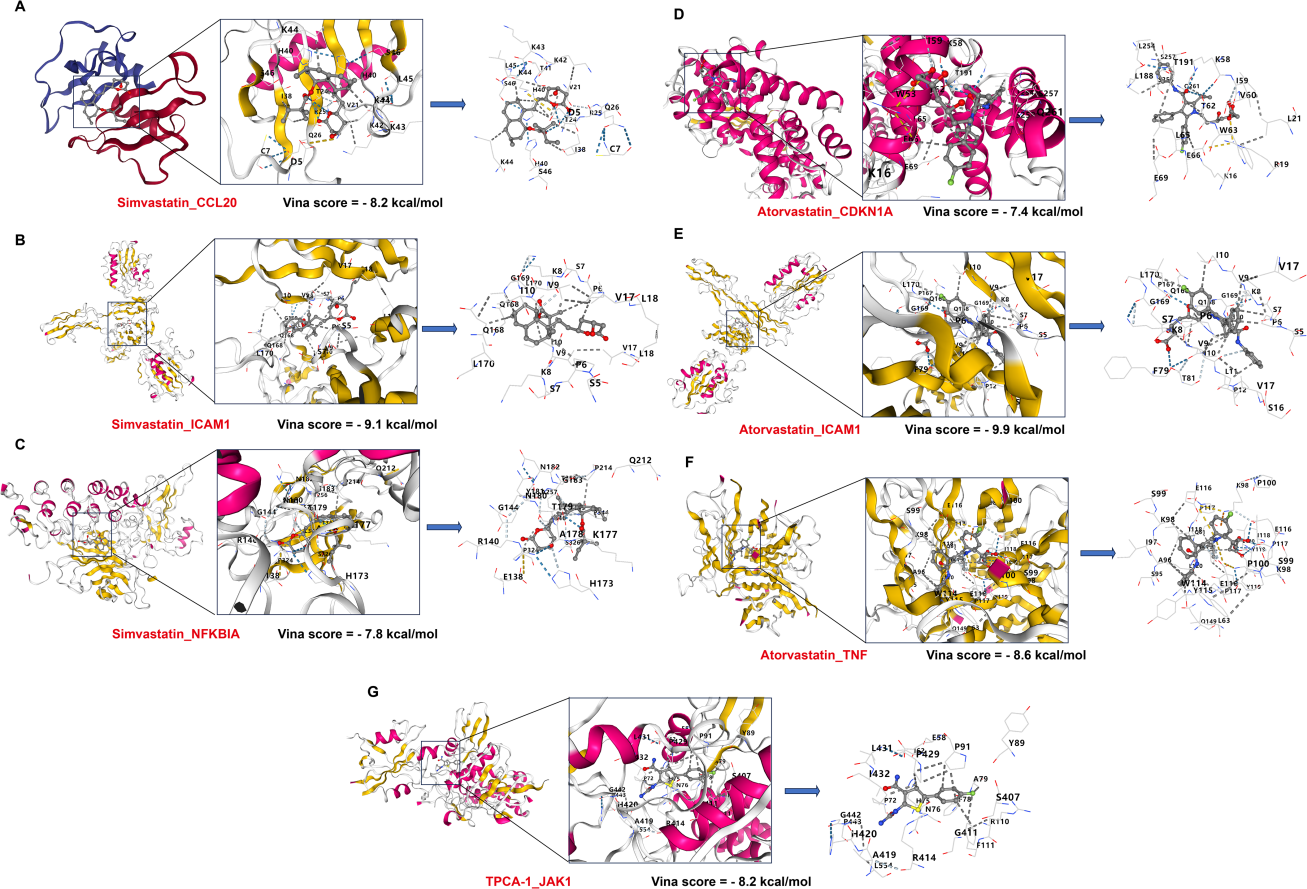
Molecular docking results of hub genes with predicted drugs. **(A–C)** Docking poses of Simvastatin with CCL20, ICAM1, and NFKBIA, with Vina scores of -8.2 kcal/mol, -9.1 kcal/mol, and -7.8 kcal/mol, respectively. **(D–F)** Docking poses of Atorvastatin with CDKN1A, ICAM1, and TNF, with Vina scores of -7.4 kcal/mol, -9.9 kcal/mol, and -8.6 kcal/mol, respectively. **(G)** Docking pose of TPCA-1with JAK1, with a Vina score of -8.2 kcal/mol. For all molecular docking diagrams, the left panel displays the binding conformation of the drug’s 3D structure with the protein, while the right enlarged panel depicts the key (or putative) binding sites between the drug and the protein.

By the CMap platform, from 10 cell types, 12 small-molecule drugs were prioritized, including: TPCA-1, cosmosiin, simvastatin, NVP-AUY922, UNC-0321, prunetin, BRD-K64835161, LY-364947, atorvastatin, tivozanib, geldanamycin, and calpeptin (**Figure 8A**). To explore interactions between hub genes and these drugs, we constructed a network using the STITCH database, revealing specific associations: Simvastatin: linked to CCL20, NFKBIA, and ICAM1; Atorvastatin: Associated with CDKN1A, ICAM1, and TNF; TPCA-1: Targeting JAK1 (**Figure 8B**). Finally, the chemical structures and 3D conformations of simvastatin, atorvastatin, and TPCA-1 were visualized using PubChem (**Figures 8C–H**). To further clarify the binding capability of hub genes to small-molecule drugs, we performed molecular docking studies using 3D structures retrieved from the RCSB Protein Data Bank and the CB-Dock platform. Results demonstrated that: Simvastatin binds tightly to CCL20, ICAM1, and NFKBIA with Vina scores of -8.2 kcal/mol, -9.1 kcal/mol, and -7.8 kcal/mol, respectively, suggesting regulatory potential (**Figures 9A–C**); Atorvastatin showed strong binding affinity to CDKN1A, ICAM1, and TNF with scores of -7.4 kcal/mol, -9.9 kcal/mol, and -8.6 kcal/mol (**Figures 9D–F**); and TPCA-1 bound to JAK1 with a score of -8.2 kcal/mol, indicating suppression of JAK1 expression ((**Figure 9G**). Collectively, these findings imply that Simvastatin, Atorvastatin, and TPCA-1 may inhibit *H. pylori*-associated GC progression by targeting hub genes (CCL20, TNF, JAK1) and modulating *H. pylori*-related inflammatory signaling pathways. These findings highlight potential therapeutic agents for modulating hub gene networks in *H. pylori*-related gastric pathogenesis, offering a foundation for experimental validation and drug repurposing strategies.

### 3.8 Experimental validation of hub genes and associated signaling pathways in *H. pylori* infection

**Figure 10:**
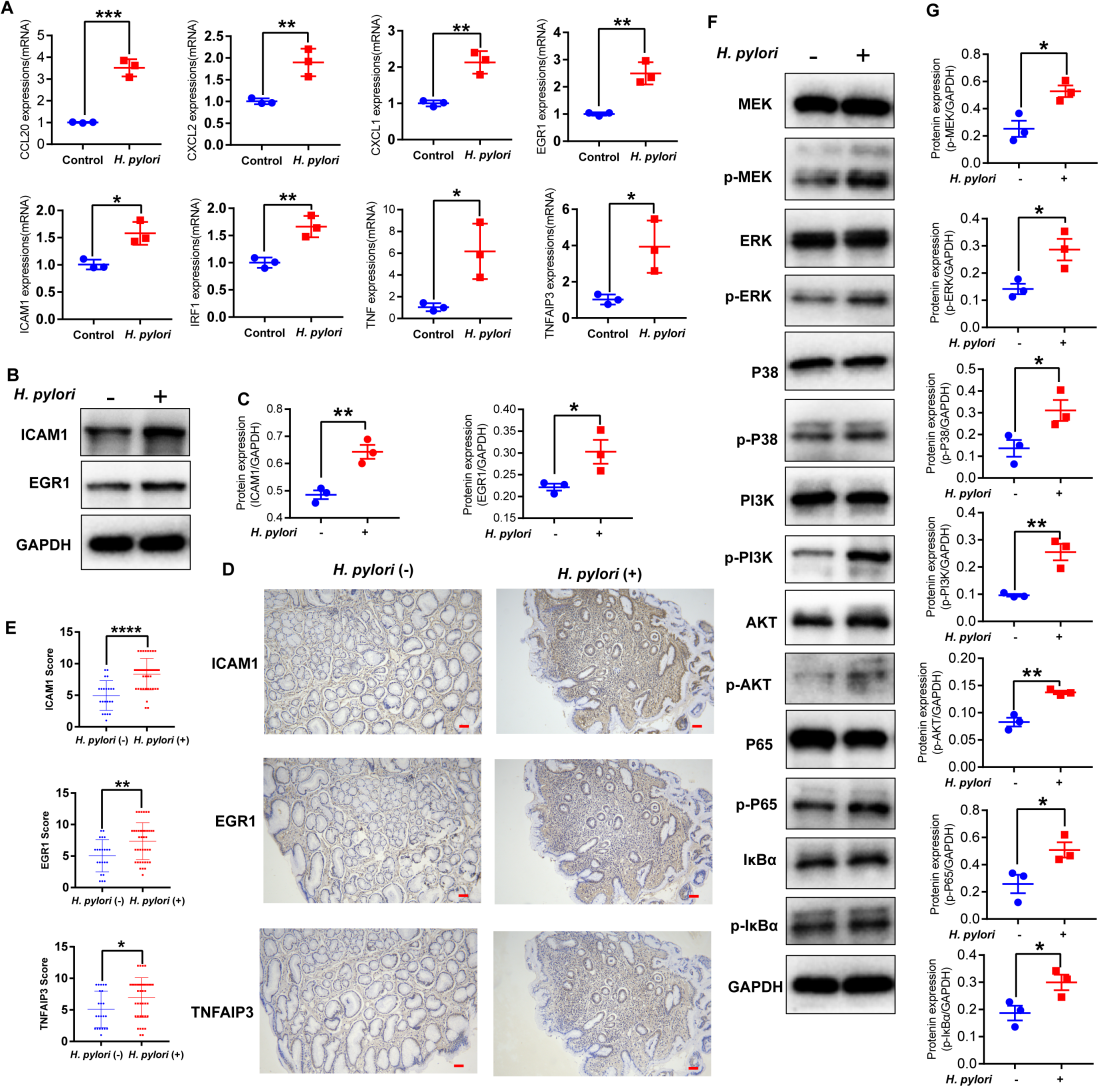
Expression of hub genes and alterations in key pathway proteins following *H. pylori* infection. **(A)** mRNA expression levels of *TNFAIP3*, *TNF*, *CXCL1*, *CXCL2*, *CCL20*, *EGR1*, *ICAM1*, and *IRF1* in AGS cells after *H. pylori* infection (MOI = 100, 24 hours; n=3). Data are presented as mean ± SD; *p < 0.05, **p < 0.01, ***p < 0.001. **(B)** WB analysis of ICAM1 and EGR1 protein expression in AGS cells infected with *H. pylori* (MOI = 100, 24 hours). GAPDH was used as the loading control. **(C)** Statistical analysis of ICAM1 and EGR1 protein expression levels, presented as the ratio of the target protein grayscale value to that of GAPDH. *p < 0.05; **p < 0.01. n=3 in each series. **(D)** IHC staining of ICAM1, EGR1, and TNFAIP3 in gastric mucosal tissues from *H. pylori*-infected and uninfected subjects. Scale bar: 50 µm. **(E)** Quantitative analysis of IHC results shown in **(D)**. *H. pylori*-negative group (n=22) *vs*. *H. pylori*-positive group (n=41); *p < 0.05, **p < 0.01, ****p < 0.0001. **(F)** WB analysis of phosphorylated signaling proteins in *H. pylori*-infected AGS cells (MOI = 100, 24 h). Key components of the MAPK/ERK pathway (p-P38, p-MEK, p-ERK), the PI3K/Akt pathway (p-PI3K, p-AKT), and the NF-κB pathway (p-P65, p-IκBα) were examined. **(G)** Quantitative analysis of the phosphorylated protein levels shown in **(F)**. Band intensities were quantified by grayscale analysis and normalized to GAPDH. Data are presented as the mean ± SD from three independent experiments. *p < 0.05, **p < 0.01. Note: The immunohistochemical images show serial paraffin sections.

Based on the aforementioned bioinformatics analysis, we identified hub genes potentially involved in *H. pylori*-induced gastric mucosal pathology and predicted their regulatory mechanisms. Subsequently, we performed *in vitro* and *in vivo* validation on selected hub genes. First, we established an *in vitro* model using *H. pylori*-infected gastric mucosal epithelial cells. At the RNA level, we observed upregulated expression of *TNFAIP3*, *TNF*, *CXCL1*, *CXCL2*, *CCL20*, *EGR1*, *ICAM1*, and *IRF1* (**Figure 10A**), which is consistent with our prior bioinformatics results. At the protein level, increased expression of EGR1 and ICAM1 was confirmed (**Figure 10B, C**). Furthermore, IHC analysis of *H. pylori*-infected gastric mucosal tissues revealed elevated expression of ICAM1, EGR1, and TNFAIP3 (**Figures 10D, E**). In line with our previous studies, which also indicated the significant upregulation and important roles of the hub genes TRAF1 and TNFAIP3 in *H. pylori*-infected gastric mucosa (Xiao et al., 2025; Zhang et al., 2025c).

In the *H. pylo*ri-infected GECs mentioned above, the MAPK, PI3K-Akt, and NF-κB signaling pathways were activated. We validated key proteins of these pathways at the protein level. The results showed increased expression of phosphorylated P38, MEK, and ERK (key proteins of the MAPK/ERK pathway), phosphorylated PI3K and AKT (key proteins of the PI3K-Akt pathway), as well as phosphorylated P65 and IκBα (key proteins of the NF-κB pathway) (**Figure 10F, G**). Additionally, our prior research demonstrated that *H. pylori* VacA modulates the TRAF1-mediated 4-1BB/NF-κB signaling axis to induce host cell apoptosis and chronic inflammatory damage (Yuan et al., 2025).

## 4 Discussion

GECs are the primary point of contact between *H. pylori* and the host. The interaction between *H. pylori* and GECs plays a critical role in the pathogenesis of *H. pylori* associated diseases, including chronic gastritis, peptic ulcers, and GC. In this study, we employed an integrative transcriptomics approach to identify and characterize pathogenic genes associated with *H. pylori* infection. To address the inherent heterogeneity of transcriptomic data, we established *H. pylori*-infected models using four gastric epithelial cell lines (HGC27, AGS, MKN45, and GES-1). Following rigorous quality control checks, RNA-seq was performed on these models. Concurrently, we retrieved RNA-seq datasets from *H. pylori*-infected clinical gastric mucosal samples in the GEO database. By integrating *in vitro* (cell-based) and *in vivo* (clinical) RNA-seq results, we identified shared gene expression patterns and potential regulatory molecular mechanisms underlying *H. pylori* infection. This dual-strategy approach minimizes model-specific biases and enhances the robustness of our findings. Our work establishes a theoretical foundation for elucidating *H. pylori*-driven pathogenic mechanisms and highlights novel targets for future therapeutic exploration.

In *H. pylori*-infected GECs (HGC27, AGS, MKN45), we identified 10 shared significantly activated signaling pathways. Studies have shown that *H. pylori* alter the expression profile of p53 protein isoforms and modulates p53-mediated responses to cellular stress (Wei et al., 2012), CagA promotes ubiquitination and proteasomal degradation of p53, suppressing its tumor-suppressive function and enhancing the survival of GECs with persistent DNA damage (Wei et al., 2010). Additionally, the initiation of *H. pylori* chromosomal replication is regulated by the interaction between the initiator protein DnaA and the regulator HobA (Terradot and Zawilak-Pawlik, 2010), *H. pylori* can mediate mitochondrial DNA replication through VacA-dependent and VacA-independent pathways (Chatre et al., 2017). *H. pylori* infection induces MAPK phosphorylation (Allison et al., 2009) and upregulates MMP-3 and MMP-9 via the MAPK signaling pathway (Karayiannis et al., 2023), Notably, CagA suppresses Runx3 expression in GECs through the Src/MEK/ERK and p38 MAPK pathways (Liu et al., 2012). *H. pylori* activates the PI3K/AKT/mTOR and NF-κB pathways, driving the expression of MMP-7 and MMP-10 (Lee et al., 2022), The CagA protein also amplifies inflammatory responses via the c-Met-PI3K/Akt-mTOR signaling axis (Li et al., 2017). IL-17 is implicated in *H. pylori*-induced gastric inflammation (Caruso et al., 2008; Shiomi et al., 2008). *H. pylori* infection activates the NLRP3 inflammasome through the TNF/TNFR1 axis, promoting M1 macrophage polarization and gastric inflammation (Fei et al., 2025). The *H. pylori*-secreted protein HP1173 induces macrophage production of TNF, CXCL8, and IL-1β via the MAP kinase, NF-κB, and AP-1 signaling pathways (Tavares and Pathak, 2018). Furthermore, *H. pylori* infection is linked to apoptosis, necroptosis, and cell cycle dysregulation in GECs (Cui et al., 2022; Ding et al., 2008; Lim et al., 2023; Yahiro et al., 2012). These findings align with our RNA-seq results, which highlight the activation of these critical pathways in *H. pylori*-infected cells, underscoring their central role in *H. pylori*-driven pathogenesis.

*H. pylori* produce metabolites such as glycine, serine, threonine, alanine, aspartate, and glutamate. Notably, glycine has been proposed as a potential therapeutic agent against *H. pylori*, particularly in combination with antibacterial drugs for treating *H. pylori*-associated diseases (Minami et al., 2004). Serine phosphorylation of cortactin regulates focal adhesion kinase activity and mediates cell scattering induced by *H. pylori* infection (Tegtmeyer et al., 2011). Additionally, *H. pylori*-derived γ-glutamyltranspeptidase (GGT) induces tolerogenic human dendritic cells by activating glutamate receptors and contributes to gastric mucosal damage (Du et al., 2020; Käbisch et al., 2016). The differential metabolic pathways identified in this study, particularly those involving glycine, serine, and glutamate metabolism, are rarely reported in *H. pylori* infection research and require further investigation to elucidate their roles in pathogenesis.

Multi-omics heterogeneity refers to biological or technical variations that exist either between different omics datasets (e.g., genome, transcriptome, proteome, metabolome) or within the same omics dataset (e.g., across samples, models, or experimental conditions). In this study, we established *H. pylori*-infected models using four gastric epithelial cell lines (HGC27, AGS, MKN45, and GES-1), performed RNA-seq, and conducted integrated bioinformatic analyses. By combining RNA-seq data from clinical *H. pylori*-infected gastric mucosal samples retrieved from the GEO database, we identified 107 co-expressed DEGs associated with *H. pylori* infection. Building on our previous findings of *H. pylori*-regulated signaling pathways, we further investigated the mechanistic roles of these DEGs. Using Cytoscape, we constructed a molecular interaction network and prioritized 20 hub genes: TNF, CXCL8, NFKBIA, IRF1, ICAM1, TNFAIP3, BIRC3, CXCL1, ITGAM, RELB, CXCL2, CCL20, NFKB2, EGR1, CDKN1A, IRAK2, JAK1, NFKBIE, TRAF1, and JUNB. Among these, the roles of TRAF1 and TNFAIP3 in *H. pylori* infection have been previously characterized (Xiao et al., 2025; Zhang et al., 2025c). Notably, our results revealed upregulated expression of NF-κB pathway-related genes (NFKBIE, NFKB2, NFKBIA), consistent with extensive evidence demonstrating *H. pylori*-induced activation of the NF-κB pathway (Cao et al., 2022; Kang et al., 2023; Maubach et al., 2022; Xie et al., 2024). Additionally, during *H. pylori* infection, its virulence factors play critical roles in disease progression. By analyzing RNA-seq data from GEO for *H. pylori* strains with cagA knockout, cagPAI mutation, vacA mutation, vacA-cagPAI double mutation, and babA mutation, we observed that knockout of these virulence factors led to reduced expression of hub genes, except for the vacA mutation (which showed increased expression). Thus, cagA, cagPAI, babA, and vacA-cagPAI are pivotal in *H. pylori* pathogenesis. Based on recent studies, we hypothesize that cagA and vacA regulate these hub genes to mediate *H. pylori*-induced pathogenicity. In future studies, we will further construct mutants of relevant *H. pylori* virulence factors and perform RNA-seq sequencing to analyze their relationship with hub genes in greater depth. These significantly upregulated hub genes in the validation set will be further analyzed to identify potential therapeutic targets for *H. pylori*-associated gastric mucosal lesions. This work lays a theoretical foundation for understanding *H. pylori*-driven molecular mechanisms and advancing precision treatment strategies.

Validation of hub genes in the GSE27411 dataset revealed significant upregulation of TNF, NFKBIA, IRF1, ICAM1, BIRC3, CXCL1, CXCL2, CCL20, NFKB2, and NFKBIE, with similar results observed in *H. pylori*-infected GES-1 cells. Further enrichment analysis linked these genes to the NF-κB pathway, lipopolysaccharide (LPS) response, bacterial defense mechanisms, IL-17 signaling, and TNF signaling. In IRF-1-deficient (IRF-1^-/-^) mice infected with *H. pylori*, severe gastritis, atrophy, and a Th1-polarized immune response were observed (Sommer et al., 2001). Studies demonstrate that interferon-stimulated response elements (ISRE), NF-κB, and activator protein (AP)-1 mediate *H. pylori*-induced IL-8 gene transcription. Additionally, OipA and the cag pathogenicity island (cagPAI) activate interferon regulatory factor (IRF)-1, which binds and activates ISRE-like elements (Yamaoka et al., 2004). *H. pylori*-derived VacA upregulates ICAM1 expression (Takeshima et al., 2009). *H. pylori* outer membrane vesicles (OMVs) induce ICAM-1 on GECs and β2 integrin CD11b on eosinophils. Eosinophil degranulation in response to *H. pylori* OMVs occurs via CD11/CD18 and ICAM-1 dependent mechanisms (Ko et al., 2015). *H. pylori* urease (HPU) triggers ROS production in endothelial cells, upregulating ICAM-1 and enhancing vascular endothelial growth factor receptor 2 (VEGFR-2) expression (de Jesus Souza et al., 2019). The EGFR inhibitor gefitinib reduces CXCL1 and CXCL2 expression in murine GECs, mitigating *H. pylori*-induced DNA damage (Sierra et al., 2018). *H. pylori* infection drives DNA methylation-based epigenetic regulation of CXCL1, with promoter hypomethylation detectable in early gastric carcinogenesis (Muhammad et al., 2023). In studies of spontaneous intracerebral hemorrhage (ICH) comorbid with *H. pylori* infection, CXCL1 and CXCL2 were also upregulated (Liu et al., 2023). While these findings highlight the central roles of hub genes in *H. pylori* related gastric mucosal diseases, their precise mechanisms remain incompletely understood and warrant further investigation.

TFs are proteins that bind specifically to DNA regulatory sequences (e.g., promoters, enhancers) to activate or repress gene transcription, thereby regulating the expression levels of target genes. In this study, we employed the TRRUST database to identify TFs potentially regulating the hub genes. Validation in clinical samples from *H. pylori*-infected patients revealed 9 significantly upregulated TFs and 4 downregulated TFs, with their expression patterns (upregulated or downregulated) visualized in the GSE233973 dataset. Notably, ETS1 was significantly upregulated in both *H. pylori*-infected clinical samples and GECs. ETS1 (V-ets erythroblastosis virus E26 oncogene homolog 1), a member of the E26 transformation-specific (ETS) family of TFs, is overexpressed in GC and associated with poor prognosis. Research has established that ETS1 plays a crucial role in promoting GC progression through a multifaceted regulatory network. Its expression is significantly upregulated in GECs of both humans and mice infected with *H. pylori*, with levels positively correlating with the severity of gastric inflammation. Mechanistically, the *H. pylori* virulence factor CagA induces ETS1 expression. This induction is further amplified as the activated transcription factor NF-κB-p65 directly upregulates ETS1, and inflammatory cytokines IL-1β and TNF-α synergistically enhance *H. pylori*−mediated ETS1 expression in an NF-κB−dependent manner (Teng et al., 2020). Once elevated, ETS1 drives GC malignancy via several downstream mechanisms. The long non-coding RNA pancEts-1 binds to NONO (non-POU domain-containing octamer-binding protein) and facilitates its physical interaction with ERG (ETS-related gene). This enhances ERG transactivation and Ets-1 transcription, creating a feed-forward loop that promotes GC progression (Li et al., 2018). Furthermore, a positive feedback circuit is formed by the lncRNA RHPN1-AS1, which acts as a competing endogenous RNA (ceRNA) to sponge miR-1299, thereby upregulating ETS1. ETS1, in turn, transcriptionally activates RHPN1-AS1, amplifying the loop to accelerate GC malignancy (Ding et al., 2020). ETS1 also contributes to metastasis under hypoxic conditions by suppressing miR-4521 expression. Conversely, miR-4521 exerts a tumor-suppressive effect by targeting IGF2 and FOXM1, which inactivates the AKT/GSK3β/Snai1 pathway and inhibits epithelial-mesenchymal transition and metastasis in GC (Xing et al., 2021). While the mechanistic role of ETS1 in *H. pylori* infection remains unclear, our TFs-hub genes interaction network visualization revealed that ETS1 regulates CDKN1A and EGR1. Furthermore, key hub genes such as TNF, CXCL8, IRF1, ICAM1, BIRC3, CXCL1, EGR1, CDKN1A, and JUNB were identified as highly interconnected nodes in the network, suggesting their central roles in *H. pylori* infection. These findings highlight potential targets for further mechanistic exploration in *H. pylori*-infected models.

In this study, we identified three small-molecule drugs linked to hub genes: Simvastatin (associated with CCL20, NFKBIA, and ICAM1), Atorvastatin (linked to CDKN1A, ICAM1, and TNF), and TPCA-1 (targeting JAK1). Clinical studies indicate that adding Simvastatin to triple therapy regimens improves *H. pylori* eradication rates (Hassan et al., 2019; Nseir et al., 2012). Simvastatin lowers cholesterol levels in GECs, attenuates CagA translocation and phosphorylation, and is associated with reduced GC risk in population cohorts (Lin et al., 2016). Atorvastatin exhibits both anti-inflammatory and antibacterial activities; its addition to quadruple therapy (omeprazole, clarithromycin, bismuth, amoxicillin) enhances *H. pylori* eradication (Sarkeshikian et al., 2020). Statins show varying bacteriostatic effects against *H. pylori*, with Atorvastatin demonstrating the strongest inhibition (Ebrahimzadeh et al., 2024). Our previous work highlighted the role of TPCA-1 in *H. pylori* infection and inflammatory bowel disease (IBD) (Zhang et al., 2024a). Moreover, we found that statins may target the STAT1–CXCL10 axis, sustain type I interferon signaling, and modulate the immune microenvironment during *H. pylori* infection (Cai et al., 2025). In GC research, knockdown of DNA damage-regulated autophagy modulator 1 (DRAM1) suppresses energy metabolism in GC cells via the PI3K/AKT/mTOR pathway, thereby slowing disease progression. Atorvastatin significantly enhances apoptosis in DRAM1-knockdown GC cells (Wu et al., 2025). Simvastatin increases the sensitivity of capecitabine by inhibiting NF-κB-regulated markers involved in proliferation, invasion, angiogenesis, and metastasis (Manu et al., 2014). Furthermore, Simvastatin reduces ILF3 expression by decreasing H3K14 acetylation at ILF3 residue sites, downregulates PD-L1 expression, activates CD8^+^ T cells, enhances antitumor immunity, and induces ferroptosis in GC cells (Sun et al., 2025). Overexpression of ITGB4 activates the MAPK-ERK-TGF-β/BMP pathway and drives endothelial-to-mesenchymal transition (EndMT) in a Smad4-dependent manner; Atorvastatin effectively blocks this ITGB4-induced EndMT by inhibiting Smad1/5 phosphorylation and promoting Smad4 ubiquitination (He et al., 2024). Atorvastatin also improves the quantity and function of bone marrow endothelial progenitor cells in patients with poor graft function by inhibiting the p38 MAPK pathway *in vitro* (Shi et al., 2016). In pancreatic cancer, the long non-coding RNA LINC00857 binds to FOXM1 and the deubiquitinase OTUB1, stabilizes FOXM1 by inhibiting its ubiquitin-proteasome degradation, and promotes metastasis; Atorvastatin suppresses this process by inhibiting the mutant p53–LINC00857 axis (Zhang et al., 2023). In summary, statins exert multifaceted effects in the context of *H. pylori* infection and GC through modulation of several signaling pathways, including the STAT1–CXCL10 axis, PI3K/AKT/mTOR, and NF-κB. Evidence from other disease models further indicates their involvement in MAPK/ERK and p53-related pathways. These findings suggest that statins may influence the pathological progression of gastric mucosa following *H. pylori* infection via multiple signaling mechanisms, the details of which merit further investigation.

In summary, Simvastatin, Atorvastatin, and TPCA-1 play critical roles in *H. pylori*-associated diseases and GC. Repurposing existing drugs to target inflammation, apoptosis, or metabolic pathways in *H. pylori* infection may improve eradication rates and reduce GC incidence. Future Directions:1) Experimental Models: *In vitro*: Treat *H. pylori*-infected GECs with candidate drugs to assess effects on inflammation, apoptosis, and bacterial load. *In vivo*: Use *H. pylori*-infected mouse models to evaluate drug impacts on mucosal inflammation, precancerous lesions, and bacterial colonization. Multi-Omics Integration: Combine transcriptomic (hub gene dynamics), metabolomic (drug metabolites), and microbiome (*H. pylori* abundance) analyses for mechanistic insights. 2) Clinical Validation: Conduct retrospective cohort studies to analyze *H. pylori* infection rates and gastritis/GC incidence in populations using repurposed drugs (e.g., statins). This integrated strategy will elucidate the mechanisms of the identified hub genes and pathways while advancing drug repurposing as a high-efficiency, low-risk therapeutic approach for *H. pylori*-related diseases, accelerating clinical translation.

While this study provides a comprehensive bioinformatics-driven characterization of transcriptomic alterations in *H. pylori*-infected gastric epithelial models, it is important to acknowledge its limitations. Primarily, our findings are largely derived from the integrative analysis of publicly available RNA-seq datasets and our *in vitro* cell line models, which may not fully capture the complexity of the human gastric mucosal microenvironment *in vivo*. The identified hub genes, regulatory networks, and predicted small-molecule drugs, though highly promising, require rigorous experimental validation. Specifically, the functional and mechanistic roles of the prioritized hub genes (e.g., TRAF1, TNFAIP3, CXCL2, CCL20, ICAM1), in *H. pylori*-driven pathogenesis, and the efficacy and specificity of the repurposed drugs (Simvastatin, Atorvastatin, TPCA-1) remain to be confirmed through *in vitro* gain- and loss-of-function studies and *in vivo* preclinical models. Furthermore, the cellular heterogeneity of the gastric epithelial lines used, while helpful for identifying shared responses, may also introduce biases that omit critical cell-type-specific mechanisms. Future work integrating multi-omics approaches (proteomics, metabolomics) with spatial transcriptomics in primary human tissues and employing genetically engineered mouse models will be essential to validate these computational predictions, elucidate causal relationships, and translate these findings into clinically actionable insights.

## 5 Conclusion

In this study, *H. pylori* infection was found to activate multiple signaling pathways, including the p53 signaling pathway, DNA replication, Apoptosis, MAPK signaling pathway, TNF signaling pathway, PI3K-Akt signaling pathway, IL-17 signaling pathway, Cell cycle, Necroptosis, and NF-kappa B signaling pathway. Using RNA-seq data from *H. pylori*-infected clinical samples and GECs, we identified 107 DEGs. Systematic screening prioritized 20 hub genes, and further validation revealed significant upregulation of TNF, NFKBIA, IRF1, ICAM1, BIRC3, CXCL1, CXCL2, CCL20, NFKB2, and NFKBIE. Importantly, the transcription factor ETS1 was identified as a critical regulator of hub genes transcription. Small-molecule drug prediction demonstrated that Simvastatin, Atorvastatin, and TPCA-1 inhibit the expression of these hub genes. We hypothesize that Atorvastatin suppresses CDKN1A and ICAM1 expression, and ETS1 may regulate CDKN1A transcription. These findings provide a theoretical foundation for understanding the pathogenic mechanisms of *H. pylori* infection and highlight potential therapeutic targets for *H. pylori*-associated diseases.

## Data Availability

The datasets presented in this study can be found in online repositories. The names of the repository/repositories and accession number(s) can be found below: https://www.ncbi.nlm.nih.gov/geo/, GSE295523.

## References

[B1] Allison C. C. Kufer T. A. Kremmer E. Kaparakis M. Ferrero R. L. (2009). Helicobacter pylori induces MAPK phosphorylation and AP-1 activation via a NOD1-dependent mechanism. J. Immunol. 183, 8099–8109. doi: 10.4049/jimmunol.0900664, PMID: 20007577

[B2] Arnold M. Park J. Y. Camargo M. C. Lunet N. Forman D. Soerjomataram I. (2020). Is gastric cancer becoming a rare disease? A global assessment of predicted incidence trends to 2035. Gut 69, 823–829. doi: 10.1136/gutjnl-2019-320234, PMID: 32001553 PMC8520492

[B3] Ashburner M. Ball C. A. Blake J. A. Botstein D. Butler H. Cherry J. M. . (2000). Gene ontology: tool for the unification of biology. The Gene Ontology Consortium. Nat. Genet. 25, 25–29. doi: 10.1038/75556, PMID: 10802651 PMC3037419

[B4] Barhoine M. Moustaoui F. Hammani O. Aghrouch M. Lemkhente Z. Belhabib Z. . (2025). The Effect of Helicobacter pylori Gene Combinations of cagA, cagE, virB11, vacA, and babA on the Outcome of Gastric Disease in a Southern Moroccan Population. Pathogens 14, 279. doi: 10.3390/pathogens14030279, PMID: 40137764 PMC11944658

[B5] Burley S. K. Bhikadiya C. Bi C. Bittrich S. Chen L. Crichlow G. V. . (2021). RCSB Protein Data Bank: powerful new tools for exploring 3D structures of biological macromolecules for basic and applied research and education in fundamental biology, biomedicine, biotechnology, bioengineering and energy sciences. Nucleic Acids Res. 49, D437–d451. doi: 10.1093/nar/gkaa1038, PMID: 33211854 PMC7779003

[B6] Cai T. Zhao X. Hu H. Wang F. Zhang M. (2025). Integrated multi-omics and molecular docking reveal shared molecular mechanisms of helicobacter pylori infection and rheumatic diseases. Int. J. Surg. doi: 10.1097/js9.0000000000003741, PMID: 41217343

[B7] Cao L. Zhu S. Lu H. Soutto M. Bhat N. Chen Z. . (2022). Helicobacter pylori-induced RASAL2 Through Activation of Nuclear Factor-κB Promotes Gastric Tumorigenesis via β-catenin Signaling Axis. Gastroenterology 162, 1716–1731.e17. doi: 10.1053/j.gastro.2022.01.046, PMID: 35134322 PMC9038683

[B8] Caruso R. Fina D. Paoluzi O. A. Del Vecchio Blanco G. Stolfi C. Rizzo A. . (2008). IL-23-mediated regulation of IL-17 production in Helicobacter pylori-infected gastric mucosa. Eur. J. Immunol. 38, 470–478. doi: 10.1002/eji.200737635, PMID: 18200634

[B9] Chatre L. Fernandes J. Michel V. Fiette L. Avé P. Arena G. . (2017). Helicobacter pylori targets mitochondrial import and components of mitochondrial DNA replication machinery through an alternative VacA-dependent and a VacA-independent mechanisms. Sci. Rep. 7, 15901. doi: 10.1038/s41598-017-15567-3, PMID: 29162845 PMC5698309

[B10] Chmiela M. Karwowska Z. Gonciarz W. Allushi B. Stączek P. (2017). Host pathogen interactions in Helicobacter pylori related gastric cancer. World J. Gastroenterol. 23, 1521–1540. doi: 10.3748/wjg.v23.i9.1521, PMID: 28321154 PMC5340805

[B11] Clyne M. Ó Cróinín T. (2025). Pathogenicity and virulence of Helicobacter pylori: A paradigm of chronic infection. Virulence 16, 2438735. doi: 10.1080/21505594.2024.2438735, PMID: 39725863 PMC12915409

[B12] Cui G. Yuan A. Li Z. (2022). Occurrences and phenotypes of RIPK3-positive gastric cells in Helicobacter pylori infected gastritis and atrophic lesions. Dig. Liver Dis. 54, 1342–1349. doi: 10.1016/j.dld.2022.04.013, PMID: 35514018

[B13] de Jesus Souza M. de Moraes J. A. Da Silva V. N. Helal-Neto E. Uberti A. F. Scopel-Guerra A. . (2019). Helicobacter pylori urease induces pro-inflammatory effects and differentiation of human endothelial cells: Cellular and molecular mechanism. Helicobacter 24, e12573. doi: 10.1111/hel.12573, PMID: 30907046

[B14] Ding L. Wang L. Li Z. Jiang X. Xu Y. Han N. (2020). The positive feedback loop of RHPN1-AS1/miR-1299/ETS1 accelerates the deterioration of gastric cancer. BioMed. Pharmacother. 124, 109848. doi: 10.1016/j.biopha.2020.109848, PMID: 31982726

[B15] Ding S. Z. Smith M. F. Jr. Goldberg J. B. (2008). Helicobacter pylori and mitogen-activated protein kinases regulate the cell cycle, proliferation and apoptosis in gastric epithelial cells. J. Gastroenterol. Hepatol. 23, e67–e78. doi: 10.1111/j.1440-1746.2007.04912.x, PMID: 18702686

[B16] Du J. Li X. H. Liu F. Li W. Q. Gong Z. C. Li Y. J. (2020). Role of the outer inflammatory protein A/cystine-glutamate transporter pathway in gastric mucosal injury induced by helicobacter pylori. Clin. Transl. Gastroenterol. 11, e00178. doi: 10.14309/ctg.0000000000000178, PMID: 32677810 PMC7263648

[B17] Ebrahimzadeh M. Asgharpour F. Shokri Shirvani J. Kazemi S. Moghadamnia A. A. (2024). Unveiling the antibacterial properties of statins: an *in vitro* study on helicobacter pylori. Adv. Pharmacol. Pharm. Sci. 2024, 6380155. doi: 10.1155/2024/6380155, PMID: 39161645 PMC11333129

[B18] Fei X. Chen S. Li L. Xu X. Wang H. Ke H. . (2025). Helicobacter pylori infection promotes M1 macrophage polarization and gastric inflammation by activation of NLRP3 inflammasome via TNF/TNFR1 axis. Cell Commun. Signal 23, 6. doi: 10.1186/s12964-024-02017-7, PMID: 39762835 PMC11705855

[B19] Ferreira L. G. Dos Santos R. N. Oliva G. Andricopulo A. D. (2015). Molecular docking and structure-based drug design strategies. Molecules 20, 13384–13421. doi: 10.3390/molecules200713384, PMID: 26205061 PMC6332083

[B20] Han H. Shim H. Shin D. Shim J. E. Ko Y. Shin J. . (2015). TRRUST: a reference database of human transcriptional regulatory interactions. Sci. Rep. 5, 11432. doi: 10.1038/srep11432, PMID: 26066708 PMC4464350

[B21] Hassan A. M. Shawky M. A. E. Mohammed A. Q. Haridy M. A. Eid K. A. (2019). Simvastatin improves the eradication rate of Helicobacter pylori: upper Egypt experience. Infect. Drug Resist. 12, 1529–1534. doi: 10.2147/idr.S202346, PMID: 31239728 PMC6556530

[B22] He Q. Li J. Tao C. Zeng C. Liu C. Zheng Z. . (2024). High glutamine increases stroke risk by inducing the endothelial-to-mesenchymal transition in moyamoya disease. MedComm (2020) 5, e525. doi: 10.1002/mco2.525, PMID: 38628905 PMC11018113

[B23] Käbisch R. Semper R. P. Wüstner S. Gerhard M. Mejías-Luque R. (2016). Helicobacter pylori γ-glutamyltranspeptidase induces tolerogenic human dendritic cells by activation of glutamate receptors. J. Immunol. 196, 4246–4252. doi: 10.4049/jimmunol.1501062, PMID: 27183641

[B24] Kanehisa M. Goto S. (2000). KEGG: kyoto encyclopedia of genes and genomes. Nucleic Acids Res. 28, 27–30. doi: 10.1093/nar/28.1.27, PMID: 10592173 PMC102409

[B25] Kang J. H. Park S. Rho J. Hong E. J. Cho Y. E. Won Y. S. . (2023). IL-17A promotes Helicobacter pylori-induced gastric carcinogenesis via interactions with IL-17RC. Gastric Cancer 26, 82–94. doi: 10.1007/s10120-022-01342-5, PMID: 36125689 PMC9813207

[B26] Karayiannis I. Martinez-Gonzalez B. Kontizas E. Kokkota A. V. Petraki K. Mentis A. . (2023). Induction of MMP-3 and MMP-9 expression during Helicobacter pylori infection via MAPK signaling pathways. Helicobacter 28, e12987. doi: 10.1111/hel.12987, PMID: 37139985

[B27] Kim S. Thiessen P. A. Bolton E. E. Chen J. Fu G. Gindulyte A. . (2016). PubChem substance and compound databases. Nucleic Acids Res. 44, D1202–D1213. doi: 10.1093/nar/gkv951, PMID: 26400175 PMC4702940

[B28] Ko S. H. Jeon J. I. Kim Y. J. Yoon H. J. Kim H. Kim N. . (2015). Helicobacter pylori outer membrane vesicle proteins induce human eosinophil degranulation via a β2 Integrin CD11/CD18- and ICAM-1-dependent mechanism. Mediators Inflammation 2015, 301716. doi: 10.1155/2015/301716, PMID: 25821353 PMC4364020

[B29] Lamb J. Crawford E. D. Peck D. Modell J. W. Blat I. C. Wrobel M. J. . (2006). The Connectivity Map: using gene-expression signatures to connect small molecules, genes, and disease. Science 313, 1929–1935. doi: 10.1126/science.1132939, PMID: 17008526

[B30] Lee J. Lim J. W. Kim H. (2022). Astaxanthin inhibits matrix metalloproteinase expression by suppressing PI3K/AKT/mTOR activation in helicobacter pylori-infected gastric epithelial cells. Nutrients 14, 3427. doi: 10.3390/nu14163427, PMID: 36014933 PMC9412703

[B31] Li D. Chen Y. Mei H. Jiao W. Song H. Ye L. . (2018). Ets-1 promoter-associated noncoding RNA regulates the NONO/ERG/Ets-1 axis to drive gastric cancer progression. Oncogene 37, 4871–4886. doi: 10.1038/s41388-018-0302-4, PMID: 29773901 PMC6117270

[B32] Li N. Tang B. Jia Y. P. Zhu P. Zhuang Y. Fang Y. . (2017). Helicobacter pylori CagA Protein Negatively Regulates Autophagy and Promotes Inflammatory Response via c-Met-PI3K/Akt-mTOR Signaling Pathway. Front. Cell Infect. Microbiol. 7. doi: 10.3389/fcimb.2017.00417, PMID: 28983474 PMC5613121

[B33] Lim M. C. C. Jantaree P. Naumann M. (2023). The conundrum of Helicobacter pylori-associated apoptosis in gastric cancer. Trends Cancer 9, 679–690. doi: 10.1016/j.trecan.2023.04.012, PMID: 37230895

[B34] Lin C. J. Liao W. C. Lin H. J. Hsu Y. M. Lin C. L. Chen Y. A. . (2016). Statins attenuate helicobacter pylori cagA translocation and reduce incidence of gastric cancer: *in vitro* and population-based case-control studies. PloS One 11, e0146432. doi: 10.1371/journal.pone.0146432, PMID: 26730715 PMC4701455

[B35] Lin M. Tu R. H. Wu S. Z. Zhong Q. Weng K. Wu Y. K. . (2024). Increased ONECUT2 induced by Helicobacter pylori promotes gastric cancer cell stemness via an AKT-related pathway. Cell Death Dis. 15, 497. doi: 10.1038/s41419-024-06885-2, PMID: 38997271 PMC11245518

[B36] Liu B. Bukhari I. Li F. Ren F. Xia X. Hu B. . (2025). Enhanced LRP8 expression induced by Helicobacter pylori drives gastric cancer progression by facilitating β-Catenin nuclear translocation. J. Adv. Res. 69, 299–312. doi: 10.1016/j.jare.2024.04.002, PMID: 38609049 PMC11954824

[B37] Liu J. Zang Y. Ma C. Wang D. Tian Z. Xu X. . (2022a). Pseudophosphatase STYX is induced by Helicobacter pylori and promotes gastric cancer progression by inhibiting FBXO31 function. Cell Death Dis. 13, 268. doi: 10.1038/s41419-022-04696-x, PMID: 35338113 PMC8956710

[B38] Liu X. Li M. Han Q. Zuo Z. Wang Q. Su D. . (2023). Exploring a shared genetic signature and immune infiltration between spontaneous intracerebral hemorrhage and Helicobacter pylori infection. Microb. Pathog. 178, 106067. doi: 10.1016/j.micpath.2023.106067, PMID: 36914055

[B39] Liu Y. Yang X. Gan J. Chen S. Xiao Z. X. Cao Y. (2022b). CB-Dock2: improved protein-ligand blind docking by integrating cavity detection, docking and homologous template fitting. Nucleic Acids Res. 50, W159–w164. doi: 10.1093/nar/gkac394, PMID: 35609983 PMC9252749

[B40] Liu Z. Li H. Huang X. Liu Q. (2024). Animal models of helicobacter pylori infection and vaccines: current status and future prospects. Helicobacter 29, e13119. doi: 10.1111/hel.13119, PMID: 39108210

[B41] Liu Z. Xu X. Chen L. Li W. Sun Y. Zeng J. . (2012). Helicobacter pylori CagA inhibits the expression of Runx3 via Src/MEK/ERK and p38 MAPK pathways in gastric epithelial cell. J. Cell Biochem. 113, 1080–1086. doi: 10.1002/jcb.23440, PMID: 22266963

[B42] Lv Y. P. Cheng P. Zhang J. Y. Mao F. Y. Teng Y. S. Liu Y. G. . (2019). Helicobacter pylori-induced matrix metallopeptidase-10 promotes gastric bacterial colonization and gastritis. Sci. Adv. 5, eaau6547. doi: 10.1126/sciadv.aau6547, PMID: 30949574 PMC6447374

[B43] Malfertheiner P. Link A. Selgrad M. (2014). Helicobacter pylori: perspectives and time trends. Nat. Rev. Gastroenterol. Hepatol. 11, 628–638. doi: 10.1038/nrgastro.2014.99, PMID: 25001975

[B44] Manu K. A. Shanmugam M. K. Li F. Chen L. Siveen K. S. Ahn K. S. . (2014). Simvastatin sensitizes human gastric cancer xenograft in nude mice to capecitabine by suppressing nuclear factor-kappa B-regulated gene products. J. Mol. Med. (Berl) 92, 267–276. doi: 10.1007/s00109-013-1095-0, PMID: 24233024

[B45] Maubach G. Vieth M. Boccellato F. Naumann M. (2022). Helicobacter pylori-induced NF-κB: trailblazer for gastric pathophysiology. Trends Mol. Med. 28, 210–222. doi: 10.1016/j.molmed.2021.12.005, PMID: 35012886

[B46] Minami M. Ando T. Hashikawa S. N. Torii K. Hasegawa T. Israel D. A. . (2004). Effect of glycine on Helicobacter pylori in *vitro*. Antimicrob. Agents Chemother. 48, 3782–3788. doi: 10.1128/aac.48.10.3782-3788.2004, PMID: 15388434 PMC521915

[B47] Muhammad J. S. Manzoor S. Cui Z. G. Khoder G. (2023). DNA methylation-mediated overexpression of CXCL1 in helicobacter pylori-induced gastric cancer: in silico- and *in vitro*-based identification of a potential biomarker for carcinogenesis. Int. J. Mol. Sci. 24, 795. doi: 10.3390/ijms24010795, PMID: 36614235 PMC9820856

[B48] Nseir W. Diab H. Mahamid M. Abu-Elheja O. Samara M. Abid A. . (2012). Randomised clinical trial: simvastatin as adjuvant therapy improves significantly the Helicobacter pylori eradication rate–a placebo-controlled study. Aliment Pharmacol. Ther. 36, 231–238. doi: 10.1111/j.1365-2036.2012.05161.x, PMID: 22646167

[B49] Pincock S. (2005). Nobel prize winners robin warren and barry marshall. Lancet 366, 1429. doi: 10.1016/s0140-6736(05)67587-3, PMID: 16243079

[B50] Sarkeshikian S. S. Ghadir M. R. Alemi F. Jalali S. M. Hormati A. Mohammadbeigi A. (2020). Atorvastatin in combination with conventional antimicrobial treatment of Helicobacter pylori eradication: A randomized controlled clinical trial. J. Gastroenterol. Hepatol. 35, 71–75. doi: 10.1111/jgh.14810, PMID: 31359499

[B51] Shi M. M. Kong Y. Song Y. Sun Y. Q. Wang Y. Zhang X. H. . (2016). Atorvastatin enhances endothelial cell function in posttransplant poor graft function. Blood 128, 2988–2999. doi: 10.1182/blood-2016-03-702803, PMID: 27769957

[B52] Shiomi S. Toriie A. Imamura S. Konishi H. Mitsufuji S. Iwakura Y. . (2008). IL-17 is involved in Helicobacter pylori-induced gastric inflammatory responses in a mouse model. Helicobacter 13, 518–524. doi: 10.1111/j.1523-5378.2008.00629.x, PMID: 19166417 PMC2631559

[B53] Sierra J. C. Asim M. Verriere T. G. Piazuelo M. B. Suarez G. Romero-Gallo J. . (2018). Epidermal growth factor receptor inhibition downregulates Helicobacter pylori-induced epithelial inflammatory responses, DNA damage and gastric carcinogenesis. Gut 67, 1247–1260. doi: 10.1136/gutjnl-2016-312888, PMID: 28473630 PMC5671361

[B54] Sommer F. Faller G. Röllinghoff M. Kirchner T. Mak T. W. Lohoff M. (2001). Lack of gastritis and of an adaptive immune response in interferon regulatory factor-1-deficient mice infected with Helicobacter pylori. Eur. J. Immunol. 31, 396–402. doi: 10.1002/1521-4141(200102)31:2<396::aid-immu396>3.0.co;2-y, PMID: 11180103

[B55] Sun D. Cui X. Yang W. Wei M. Yan Z. Zhang M. . (2025). Simvastatin inhibits PD-L1 via ILF3 to induce ferroptosis in gastric cancer cells. Cell Death Dis. 16, 208. doi: 10.1038/s41419-025-07562-8, PMID: 40140647 PMC11947124

[B56] Takeshima E. Tomimori K. Takamatsu R. Ishikawa C. Kinjo F. Hirayama T. . (2009). Helicobacter pylori VacA activates NF-kappaB in T cells via the classical but not alternative pathway. Helicobacter 14, 271–279. doi: 10.1111/j.1523-5378.2009.00683.x, PMID: 19674131

[B57] Tavares R. Pathak S. K. (2018). Induction of TNF, CXCL8 and IL-1β in macrophages by Helicobacter pylori secreted protein HP1173 occurs via MAP-kinases, NF-κB and AP-1 signaling pathways. Microb. Pathog. 125, 295–305. doi: 10.1016/j.micpath.2018.09.037, PMID: 30267894

[B58] Tegtmeyer N. Wittelsberger R. Hartig R. Wessler S. Martinez-Quiles N. Backert S. (2011). Serine phosphorylation of cortactin controls focal adhesion kinase activity and cell scattering induced by Helicobacter pylori. Cell Host Microbe 9, 520–531. doi: 10.1016/j.chom.2011.05.007, PMID: 21669400

[B59] Teng Y. Cang B. Mao F. Chen W. Cheng P. Peng L. . (2020). Expression of ETS1 in gastric epithelial cells positively regulate inflammatory response in Helicobacter pylori-associated gastritis. Cell Death Dis. 11, 498. doi: 10.1038/s41419-020-2705-8, PMID: 32612120 PMC7329872

[B60] Teng Y. Xie R. Xu J. Wang P. Chen W. Shan Z. . (2023). Tubulointerstitial nephritis antigen-like 1 is a novel matricellular protein that promotes gastric bacterial colonization and gastritis in the setting of Helicobacter pylori infection. Cell Mol. Immunol. 20, 924–940. doi: 10.1038/s41423-023-01055-4, PMID: 37336990 PMC10387474

[B61] Terradot L. Zawilak-Pawlik A. (2010). Structural insight into Helicobacter pylori DNA replication initiation. Gut Microbes 1, 330–334. doi: 10.4161/gmic.1.5.13115, PMID: 21327042 PMC3023618

[B62] Warren J. R. Marshall B. (1983). Unidentified curved bacilli on gastric epithelium in active chronic gastritis. Lancet 1, 1273–1275. 6134060

[B63] Wei J. Nagy T. A. Vilgelm A. Zaika E. Ogden S. R. Romero-Gallo J. . (2010). Regulation of p53 tumor suppressor by Helicobacter pylori in gastric epithelial cells. Gastroenterology 139, 1333–1343. doi: 10.1053/j.gastro.2010.06.018, PMID: 20547161 PMC2949494

[B64] Wei J. Noto J. Zaika E. Romero-Gallo J. Correa P. El-Rifai W. . (2012). Pathogenic bacterium Helicobacter pylori alters the expression profile of p53 protein isoforms and p53 response to cellular stresses. Proc. Natl. Acad. Sci. U.S.A. 109, E2543–E2550. doi: 10.1073/pnas.1205664109, PMID: 22927405 PMC3458371

[B65] Wu X. Li Y. Wang W. Xu J. Zhao B. Sun W. . (2025). DRAM1 enhances the proliferation and metastasis of gastric cancer through the PI3K/AKT/mTOR signaling pathway and energy metabolism. Sci. Rep. 15, 3542. doi: 10.1038/s41598-025-87389-7, PMID: 39875526 PMC11775094

[B66] Xiao F. Huang G. Yuan G. Li S. Wang Y. Tan Z. . (2024). Identification and validation of potential diagnostic signature and immune cell infiltration for HIRI based on cuproptosis-related genes through bioinformatics analysis and machine learning. Front. Immunol. 15. doi: 10.3389/fimmu.2024.1372441, PMID: 38690269 PMC11058647

[B67] Xiao S. Shen Y. Zhang M. Liu X. Cai T. Wang F. (2025). VacA promotes pyroptosis via TNFAIP3/TRAF1 signaling to induce onset of atrophic gastritis. Microbiol. Res. 296, 128142. doi: 10.1016/j.micres.2025.128142, PMID: 40138873

[B68] Xie R. You N. Chen W. Y. Zhu P. Wang P. Lv Y. P. . (2024). Helicobacter pylori-induced angiopoietin-like 4 promotes gastric bacterial colonization and gastritis. Res. (Wash D C) 7, 409. doi: 10.34133/research.0409, PMID: 39022746 PMC11254415

[B69] Xing S. Tian Z. Zheng W. Yang W. Du N. Gu Y. . (2021). Hypoxia downregulated miR-4521 suppresses gastric carcinoma progression through regulation of IGF2 and FOXM1. Mol. Cancer 20, 9. doi: 10.1186/s12943-020-01295-2, PMID: 33407516 PMC7786912

[B70] Yahiro K. Satoh M. Nakano M. Hisatsune J. Isomoto H. Sap J. . (2012). Low-density lipoprotein receptor-related protein-1 (LRP1) mediates autophagy and apoptosis caused by Helicobacter pylori VacA. J. Biol. Chem. 287, 31104–31115. doi: 10.1074/jbc.M112.387498, PMID: 22822085 PMC3438942

[B71] Yamaoka Y. Kudo T. Lu H. Casola A. Brasier A. R. Graham D. Y. (2004). Role of interferon-stimulated responsive element-like element in interleukin-8 promoter in Helicobacter pylori infection. Gastroenterology 126, 1030–1043. doi: 10.1053/j.gastro.2003.12.048, PMID: 15057743

[B72] Yu Y. Yang Y. L. Chen X. Y. Chen Z. Y. Zhu J. S. Zhang J. (2024). Helicobacter pylori-enhanced hnRNPA2B1 coordinates with PABPC1 to promote non-m(6)A translation and gastric cancer progression. Adv. Sci. (Weinh) 11, e2309712. doi: 10.1002/advs.202309712, PMID: 38887155 PMC11321670

[B73] Yuan L. Yao S. Qu N. Li X. Yang X. Deng A. . (2025). Helicobacter pylori VacA modulates TRAF1-mediated 4-1BB/NF-kappaB axis to induce host apoptosis and chronic inflammatory damage. Mol. Med. 31, 317. doi: 10.1186/s10020-025-01349-5, PMID: 41136917 PMC12551314

[B74] Zhang M. Liu T. Luo L. Xie Y. Wang F. (2025a). Biological characteristics, immune infiltration and drug prediction of PANoptosis related genes and possible regulatory mechanisms in inflammatory bowel disease. Sci. Rep. 15, 2033. doi: 10.1038/s41598-024-84911-1, PMID: 39814753 PMC11736032

[B75] Zhang M. Liu T. Luo L. Zhang Y. Chen Q. Wang F. . (2024a). Common diagnostic biomarkers and molecular mechanisms of Helicobacter pylori infection and inflammatory bowel disease. Front. Immunol. 15. doi: 10.3389/fimmu.2024.1492810, PMID: 39712025 PMC11659760

[B76] Zhang M. Ma Z. Yi Z. Wang H. Zhu J. Wen G. . (2024b). SLC26A9 promotes colorectal tumorigenesis by modulating Wnt/β-catenin signaling. Cell Death Discov. 10, 123. doi: 10.1038/s41420-024-01888-6, PMID: 38461207 PMC10925040

[B77] Zhang W. Qian W. Gu J. Gong M. Zhang W. Zhang S. . (2023). Mutant p53 driven-LINC00857, a protein scaffold between FOXM1 and deubiquitinase OTUB1, promotes the metastasis of pancreatic cancer. Cancer Lett. 552, 215976. doi: 10.1016/j.canlet.2022.215976, PMID: 36272615

[B78] Zhang M. Xie J. Yao S. Cai T. Yuan L. Liu X. . (2025b). The expression signature, prognostic significance and immune cell infiltration of the OAS gene family in gastric cancer. Sci. Rep. 15, 39682. doi: 10.1038/s41598-025-23277-4, PMID: 41224952 PMC12612043

[B79] Zhang M. Yuan L. Yang X. Zhao X. Xie J. Liu X. . (2025c). TRAF1 promotes the progression of Helicobacter pylori-associated gastric cancer through EGFR/STAT/OAS signalling. Life Sci. 373, 123656. doi: 10.1016/j.lfs.2025.123656, PMID: 40287055

[B80] Zhou N. Peng L. Luo Q. Yin T. Sun H. Zhang Y. . (2025). FerrDb V3: expanding the manually curated resource for regulators and disease associations from ferroptosis to regulated cell death. Nucleic Acids Res. 54, D572–D582. doi: 10.1093/nar/gkaf1119, PMID: 41171133 PMC12807728

