# Supplementary material for: Integrated transcriptomics and molecular docking identify hub genes and statin regulators in Helicobacter pylori-associated gastric mucosal pathogenesis
Source: Front Cell Infect Microbiol. 2026 Feb 13;16:1688009. doi: 10.3389/fcimb.2026.1688009 (PMC12945777; doi:10.3389/fcimb.2026.1688009)
Supplement: Supplementary file 1 [file DataSheet1.pdf]

Supplementary Figure

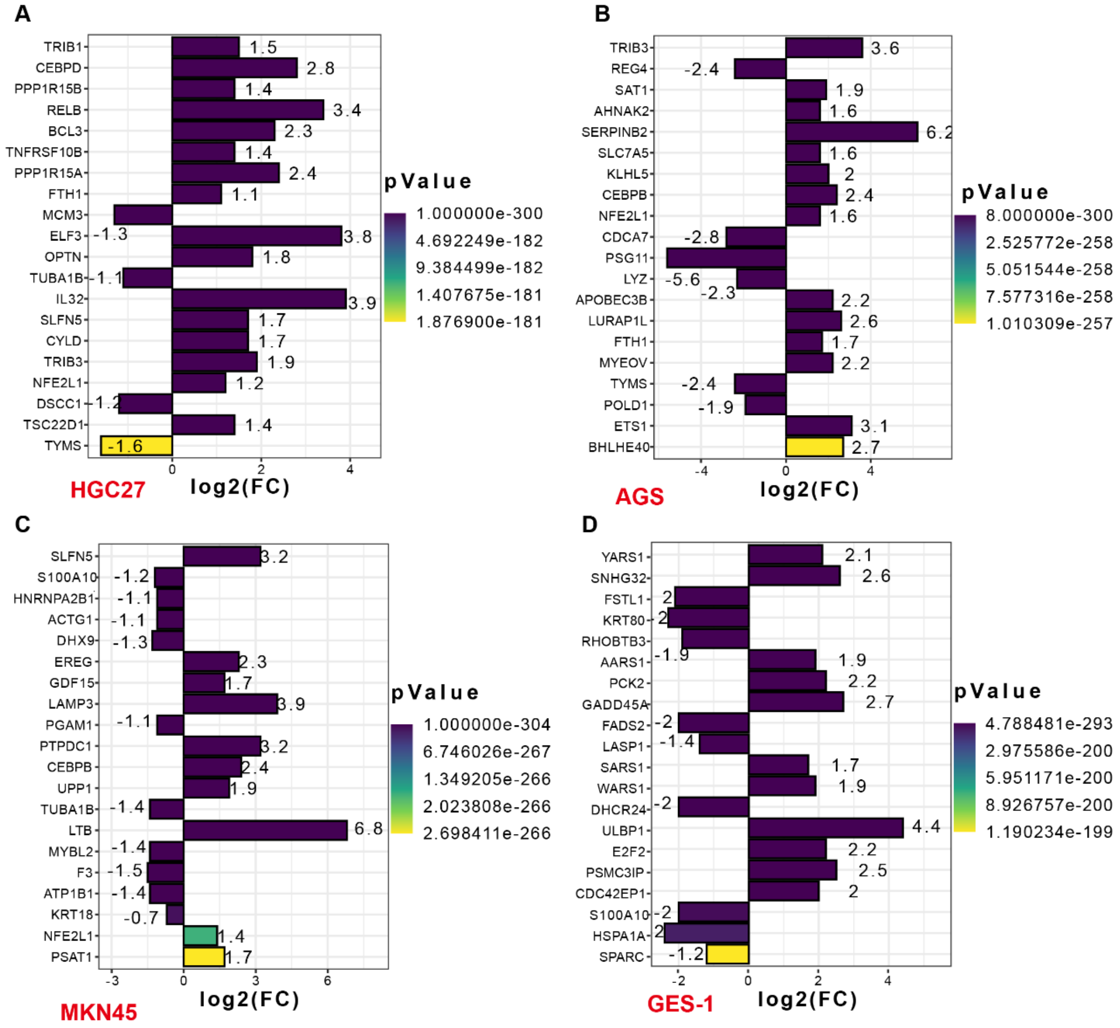

Supplementary Figure 1 Bar graphs of the TOP20 DEGs. (A-D) DEGs identified by RNA-seq in *H. pylori*-infected gastric epithelial cells (HGC27, AGS, MKN45, GES-1) at MOI=100 after 24-hour infection. These panels represent the bar graph visualization of Figure 1. X-axis: $\log_2$(FC); Left annotation: Gene names; Right annotation: p-value.

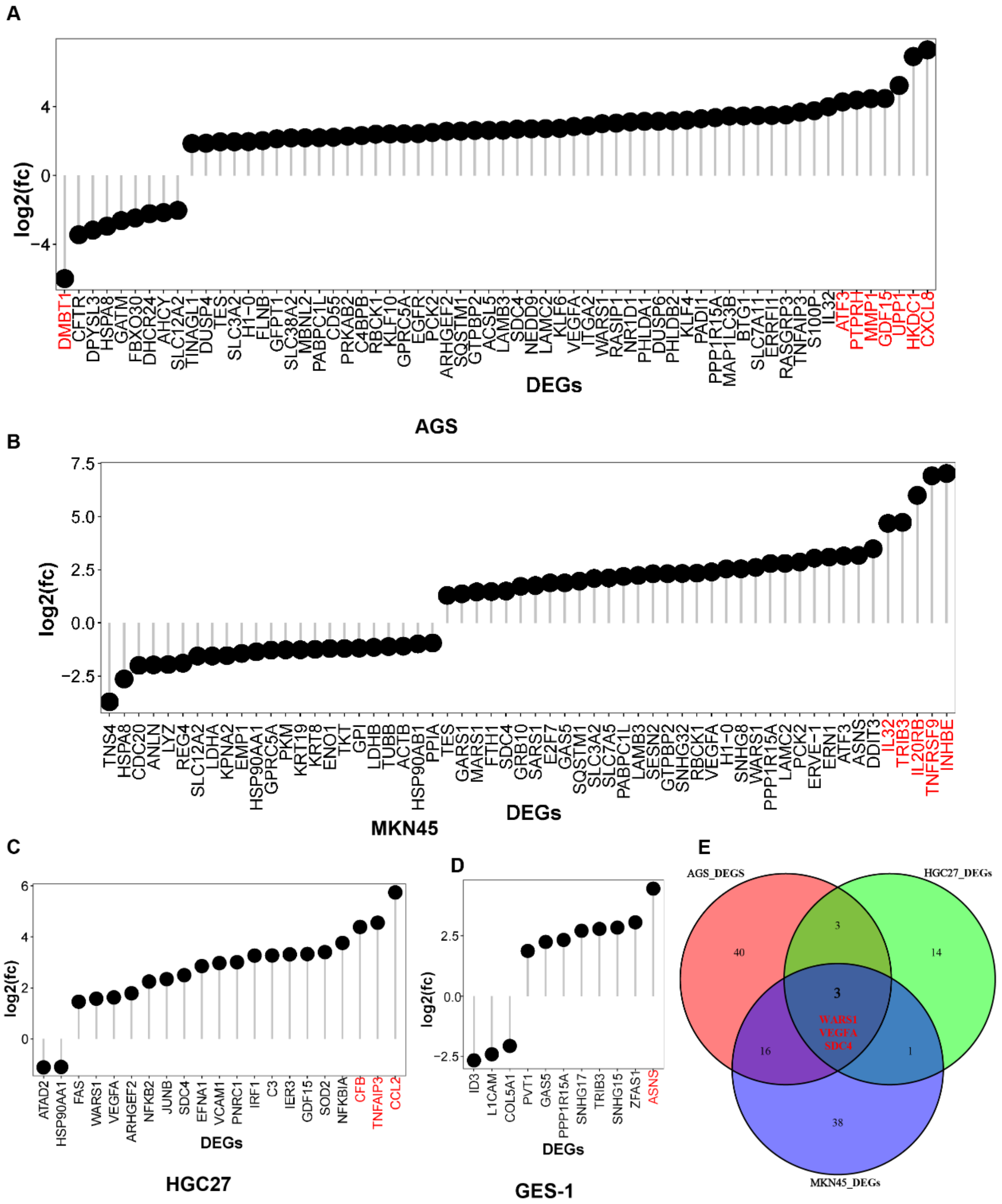

Supplementary Figure 2 Lollipop plots and venn diagram of DEGs. (A-D) Lollipop plots of RNA-seq-derived DEGs in *H. pylori*-infected AGS, MKN45, HGC27, and GES-1 cells (q-value ≈ 0, approaching zero). X-axis: Gene names; Y-axis: $\log_2$(fc). Genes with $|\log_2 FC| \geq 4$ are highlighted in red. (E) Venn diagram showing overlapping DEGs across *H. pylori*-infected HGC27, AGS, and MKN45 cells. Shared genes: *WARS1*, *VEGFA*, and *SDC4*.

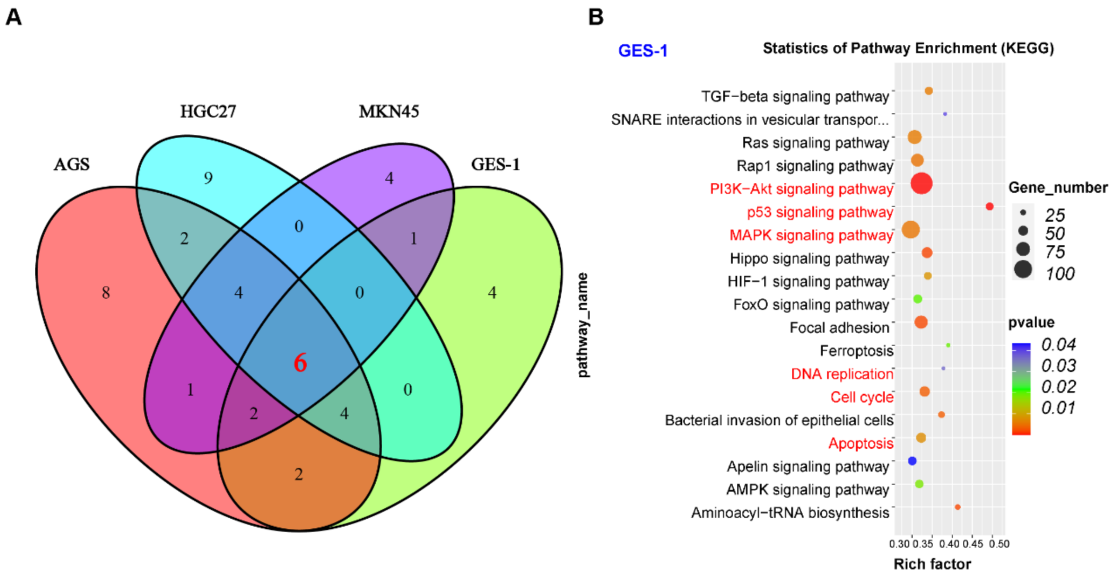

Supplementary Figure 3 KEGG pathway enrichment in *H. pylori*-infected GES-1 cells. (A) Venn diagram showing six overlapping KEGG pathways enriched across four *H. pylori*-infected gastric epithelial cell lines (HGC27, AGS, MKN45, GES-1). (B) Significantly enriched KEGG pathways in *H. pylori*-infected GES-1 cells. Pathways highlighted in red represent the six overlapping pathways shared among the four cell lines.

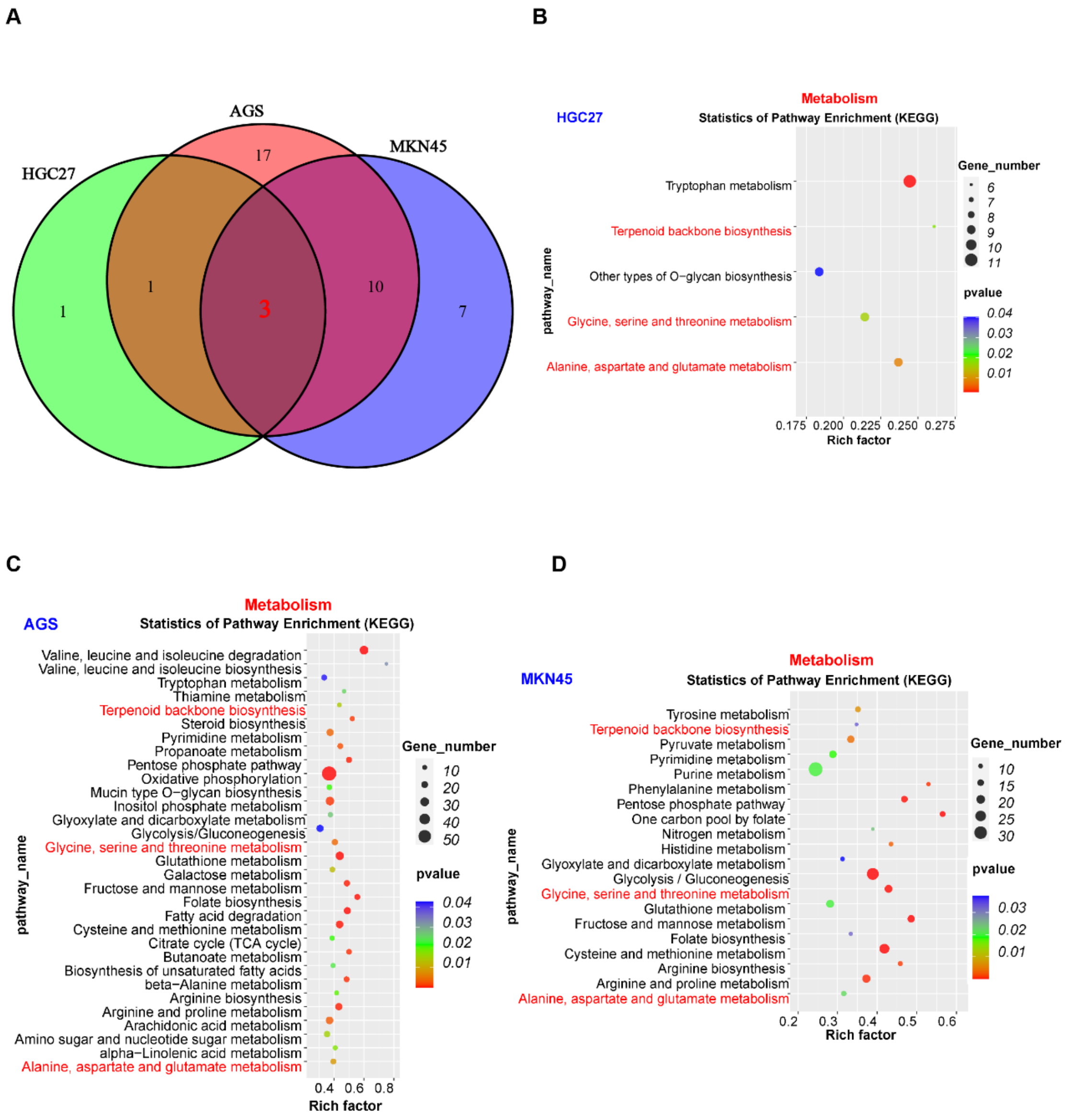

Supplementary Figure 4 KEGG metabolic pathway enrichment in *H. pylori*-infected HGC27, AGS, and MKN45 Cells. (A) Venn diagram showing three overlapping KEGG metabolic pathways enriched in *H. pylori*-infected HGC27, AGS, and MKN45 cells. (B-D) Enrichment results of significantly enriched KEGG metabolic pathways in *H. pylori*-infected HGC27 (B), AGS (C), and MKN45 (D) cells. Pathways highlighted in red represent the three overlapping metabolic pathways shared across all three cell lines.

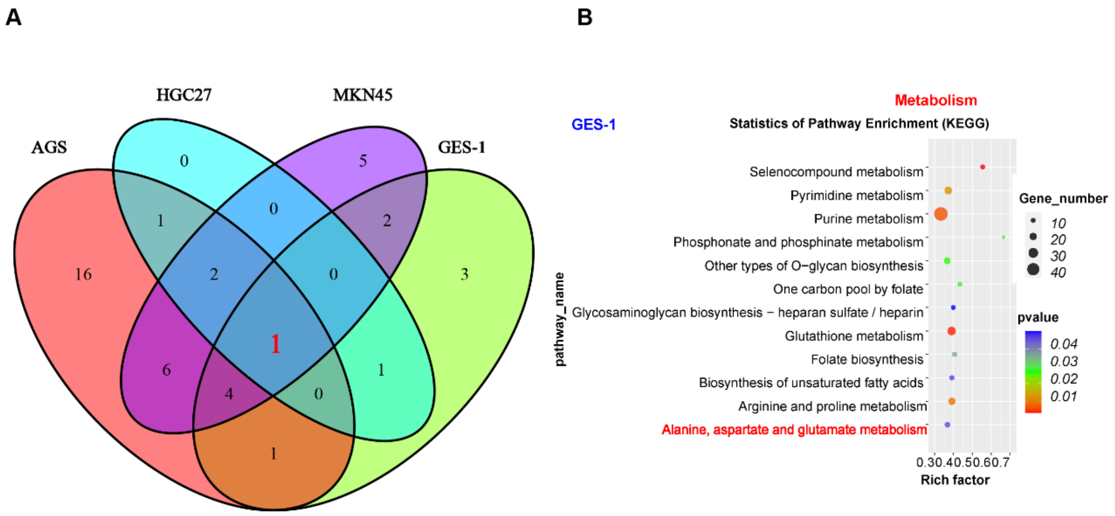

Supplementary Figure 5 KEGG metabolic pathway enrichment in *H. pylori*-infected GES-1 Cells. (A) Venn diagram of KEGG metabolic pathways enriched across four *H. pylori*-infected gastric epithelial cell lines (HGC27, AGS, MKN45, GES-1). (B) Significantly enriched KEGG metabolic pathways in *H. pylori*-infected GES-1 cells. The pathway highlighted in red represents the one overlapping metabolic pathway shared among all four cell lines.

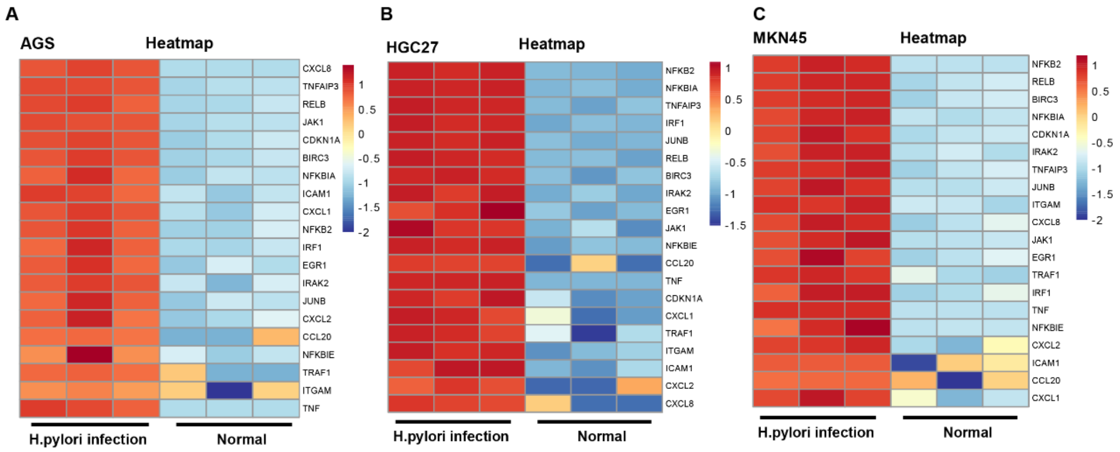

Supplementary Figure 6 Heat map of Hub genes expression. (A-C) Heatmap of Hub gene expression in *H. pylori*-infected gastric epithelial cells (HGC27, AGS, MKN45): *H. pylori* infection vs. Normal.

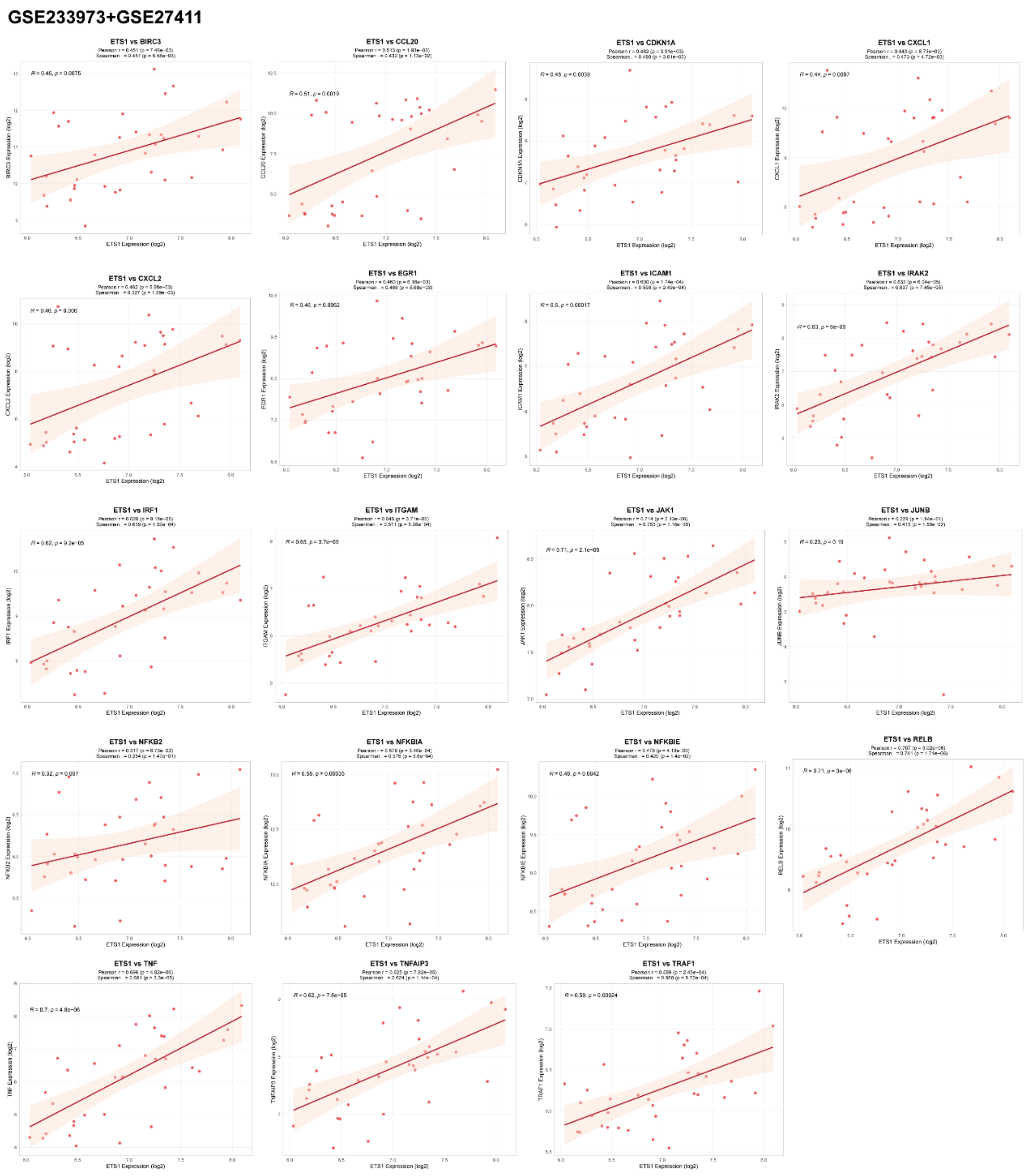

Supplementary Figure 7 Correlation analysis between ETS1 and hub genes in the combined GSE233973 and GSE27411 datasets. Scatter plots showing the expression correlation of ETS1 and hub genes. The Pearson correlation coefficient (R) and p-value are indicated for each pair. Positive correlations are observed between ETS1 and hub genes.

Supplementary Table 1. List of online public databases.

Database	Online link
TCGA database	https://portal.gdc.cancer.gov/
GEO database	https://www.ncbi.nlm.nih.gov/geo/
The Human Autophagy Database	https://www.autophagy.lu/
Ferroptosis FerrDb	http://www.zhounan.org/ferrdb/v3
PubChem	https://pubchem.ncbi.nlm.nih.gov
RCSB Protein Data Bank	https://www.rcsb.org/
CB-Dock	http://clab.labshare.cn/cb-dock/
STRING database	https://string-db.org/

Supplementary Table 2. List of primers of multiple genes was used in this study

**RT-qPCR**		
**Gene (Human)**	**Forward sequence**	**Reverse sequence**
CCL20	GGATTTGCGCACACAGACAA	GGCGAATCAGAAGCAGCAAG
TNF	CTTCCAGCTGGAGAAGGGTG	CCCAAAGTAGACCTGCCCAG
EGR1	CTGACCAAGCTGAAGAGGGG	GAGAAGGTGCTGGTGGAGAC
IRF1	TGGGGATTCCAGCCCTGATA	CACCTCCAAGTCCTGCATGT
CXCL1	AGTGTGAACGTGAAGTCCCC	ATGCAGGATTGAGGCAAGCT
TNFAIP3	AACATTTTGCTGCTGCCT	ATGGTGCTTCCAAGGGT
CXCL2	GCTGCTGCTCCTGCTCCTG	GGGACTTCACCTTCACACTTTGG
ICAM1	ACCTATGGCAACGACTCCTTCTC	GTGTCTCCTGGCTCTGGTTCC
GAPDH	AACGGATTTGGTCGTATTGGG	TCGCTCCTGGAAGATGGTGAT

Supplementary Table 3. List of antibodies for WB and IHC

Antibody	Vendor	Antigen dilution
**Primary Antibodies to**:		
ICAM-1	Proteintech	1:3000; 1:500*
EGR1	Proteintech	1:1000; 1:50*
TNFAIP3	Santa Cruz	1:1000; 1:20*
MEK	Abmart	1:1000
p-MEK	Abmart	1:1000
ERK	Abmart	1:1000
p-ERK	Abmart	1:1000
P38	Abmart	1:1000
p-P38	Abmart	1:1000
PI3K	Abmart	1:1000
p-PI3K	Abmart	1:1000
AKT	Abmart	1:1000
p-AKT	Abmart	1:1000
IκBα	Abmart	1:1000
p-IκBα	Abmart	1:1000
P65	CST	1:1000
p-P65	CST	1:1000
GAPDH	Proteintech	1:50000
Goat anti-rabbit IgG-HRP	Proteintech	1:5000
Goat anti-mouse IgG-HRP	Proteintech	1:5000

List of antibodies was used in this study, * For IHC, others for Western Blot, CST: Cell Signaling Technology.
